# Rapid and modular workflows for same-day sequencing-based detection of bloodstream infections and antimicrobial resistance determinants using culture-enriched samples

**DOI:** 10.1128/spectrum.03240-25

**Published:** 2026-06-15

**Authors:** Mohammad Saiful Islam Sajib, Katarina Oravcova, Kirstyn Brunker, Paul Everest, Manuel Fuentes, Catherine Wilson, Michael E. Murphy, Taya Forde

**Affiliations:** 1 School of Biodiversity, One Health & Veterinary Medicine, University of Glasgow 3526, Glasgow, United Kingdom; 2 MRC-University of Glasgow Centre for Virus Research 155698, Glasgow, United Kingdom; 3 Department of Microbiology, NHS Greater Glasgow and Clyde, Glasgow Royal Infirmary 3529, Glasgow, United Kingdom; 4 School of Medicine, Dentistry & Nursing, College of Medical, Veterinary & Life Sciences, Wolfson Medical School Building, University of Glasgow 12194, Glasgow, United Kingdom; Institut National de Santé Publique du Québec, Sainte-Anne-de-Bellevue, Québec, Canada; University of Edinburgh, Edinburgh, United Kingdom

**Keywords:** rapid diagnosis, selective host depletion, chemical host depletion, metagenomic next-generation sequencing, Oxford Nanopore Technologies, selective sequencing, adaptive sampling, bloodstream infection, antimicrobial resistance

## Abstract

Bloodstream infections (BSI) are a major global health concern, and existing diagnostic methods are too slow to guide targeted antibiotic therapy for critically ill patients. Rapid metagenomic next-generation sequencing (mNGS) can facilitate swift microbiological diagnosis, but identification is challenged by significant host versus bacterial DNA in blood and blood culture media. To accelerate reporting time, we developed M-15, a rapid mNGS-based host DNA depletion workflow optimized for culture-enriched samples, validated with suspected BSI blood culture samples and rapid culture-enriched spiked blood. M-15 was benchmarked with five commercial/published protocols, combined with rapid mNGS, and tested on blood culture samples (*n* = 33) from suspected BSI cases identified on BACT/ALERT-VIRTUO. To determine whether it is possible to utilize M-15 mNGS prior to blood culture flagging positive, a rapid enrichment method was tested starting with 1–10 colony-forming units of the top 15 bacterial species causing BSI spiked into BACT/ALERT medium enriched with 10 mL sheep blood. All six chemical depletion protocols reduced host DNA by 2.5 × 10^0^- to 4.1 × 10^6^-fold, with the in-house M-15 protocol performing best. With BACT/ALERT specimens, M-15 mNGS identified 28/28 mono-bacterial and 2/4 multi-bacterial species. With rapid culture enrichment and M-15 mNGS, <18% DNA was classified as host, and all bacterial species tested (*n* = 10) were correctly identified. M-15 mNGS accurately predicted phenotypic AMR/susceptibility for 90.3% (232/257) of drug/bacteria combinations from BACT/ALERT-positive samples. This study demonstrates that M-15 mNGS can facilitate species and AMR gene detection within 5–7 hours of BACT/ALERT positivity and possibly 13–15 hours of sample collection. Further clinical validation is required to assess its performance and the potential to improve patient outcomes in BSI.

**IMPORTANCE:**

Bloodstream infections (BSI) are among the leading global health challenges, and traditional culture-based diagnostic methods are too slow (often taking >48 hours) to guide critical clinical interventions. This study demonstrates the development and utility of M-15 metagenomic next-generation sequencing (mNGS), a modular Oxford Nanopore-based chemical host DNA depletion and metagenomic sequencing workflow applied to enriched blood culture media for the same-day detection of bacterial etiologies and their antimicrobial resistance (AMR) genes. The selective chemical host DNA depletion method (M-15) described in this study can remove approximately 4.1 × 10^6^-fold unwanted host DNA from whole blood, providing high-resolution genomic information from the bacteria at a fraction of the sequencing time/cost (approximately £120–£160/sample). We have tested this workflow on culture-positive clinical and rapid enriched spiked blood samples and demonstrated its ability to identify bacterial species and AMR genes between 5 and 7 hours post blood culture positivity. Based on our *in vitro* experiments using rapid enrichment, we believe similar results could be achieved within 13–15 hours from blood sample collection. Although further clinical validation is required, especially to fully assess the rapid version of the protocol, M-15 mNGS offers a promising advancement in BSI diagnosis. This workflow is modular and can be expanded in the future to adapt for other infections, which makes it a versatile tool to improve patient outcomes in sepsis.

## INTRODUCTION

Bloodstream infection (BSI) is one of the leading causes of death worldwide, with 2.9 million cases each year (1). Rapid identification of the causative bacterial pathogens and their antimicrobial resistance (AMR) profiles is crucial for guiding more effective treatment and better patient outcomes, especially for critically ill patients such as those who develop sepsis (2). Blood culture and culture-based phenotypic antimicrobial susceptibility testing (AST), the gold standard diagnostic method, often takes 48 –72 hours, which is too slow to guide targeted antibiotic therapy. This necessitates the prescription of empirical antibiotics, which may fail to cover the resistance profile of the causative pathogen and risk poor clinical outcomes (3). Also, the use of broad-spectrum antibiotics to limit disease progression may disrupt beneficial gut microbiota and contribute to the development of multidrug-resistant bacteria often associated with healthcare infections, further limiting the reserve of critically important antimicrobial agents (4). Thus, there is an urgent need for rapid diagnostic tools to better inform antibiotic regimens at the early stages of BSI.

Certain applications of metagenomic next-generation sequencing (mNGS) could be used to rapidly identify bacterial species and AMR determinants and therefore have the potential to accelerate BSI diagnosis (5). However, the proportion of host DNA in blood compared to that of bacteria makes it challenging to directly sequence and recover adequate reads from the pathogen. This makes it difficult to accurately identify species and AMR in a cost-effective manner and within a clinically relevant timeframe. To address this issue, we developed a rapid mNGS-based chemical host DNA depletion (CHDD) protocol, named M-15, and compared its performance with two commercial kits (C1–2) and three published methods (P1–3) (6–8). Next, M-15 mNGS was validated with and without nanopore adaptive sampling (a real-time selective sequencing method unique to Oxford Nanopore Technologies) using both culture (BACT/ALERT, an automated blood culture system) positive and negative enriched blood samples collected from patients with suspected BSI. Finally, M-15 was combined and tested with a rapid (8 hour) culture (BACT/ALERT) enrichment step to reduce turnaround time and enable same-day microbiological diagnosis of bloodstream infections.

## MATERIALS AND METHODS

### Bacterial strains and host matrix

Defibrinated sheep blood (E&O Laboratories, UK) and EDTA-treated whole blood from healthy human volunteers were used as model host environment matrices. The clinical and/or American Type Culture Collection (ATCC) strains tested/used in this study are described in the Results in Table 1 and Tables S1 to S3.

**TABLE 1:** Host depletion efficiency and bacterial DNA loss observed with in-house chemical host DNA depletion method, M-15, compared with two commercial kits (C1–2) and three published protocols (P1–3)^*a*^

Organism	Methods/Kits	No chemical depletion Ct (SD)	Chemical depletion Ct (SD)	Mean ΔCt	Mean fold reduction
*Ovis aries*	M-15 (this study)	16.24 (0.79)	36.76 (1.82)	−20.52	1.5 × 10^6^***
*Ovis aries*	Molysis Complete 5 (C2)	20.51 (0.64)	25.73 (1.08)	−5.22	3.7 × 10^1^***
*Ovis aries*	P1 (6)	16.24 (0.79)	33.69 (0.99)	−17.45	1.8 × 10^5^***
*Ovis aries*	P2 (7)	18.97 (0.49)	21.15 (0.53)	−2.18	4.5 × 10^0^***
*Ovis aries*	P3 (8)	18.97 (0.49)	20.15 (0.51)	−1.18	2.3 × 10^0^***
*Ovis aries*	Zymo-HostZERO (C1)	20.30 (0.58)	29.74 (2.10)	−9.44	6.9 × 10^2^***
*Escherichia coli*	M-15 (this study)	23.69 (1.04)	23.91 (0.47)	−0.22	1.2 × 10^0^ (NS)
*E. coli*	Molysis Complete 5 (C2)	23.73 (0.67)	23.27 (0.55)	0.46	7.3 × 10^−1^ (NS)
*E. coli*	P1 (6)	23.94 (0.43)	22.41 (0.22)	1.53	3.5 × 10^−1^ (NS)
*E. coli*	P2 (7)	22.18 (0.28)	22.84 (0.73)	−0.66	1.6 × 10^0^*
*E. coli*	P3 (8)	22.18 (0.28)	21.39 (0.24)	0.79	5.8 × 10^−1^ (NS)
*E. coli*	Zymo-HostZERO (C1)	24.57 (0.34)	25.34 (0.65)	−0.77	1.7 × 10^0^**
*Pseudomonas aeruginosa*	M-15 (this study)	23.09 (0.46)	24.26 (0.66)	−1.17	2.3 × 10^0^***
*P. aeruginosa*	Molysis Complete 5 (C2)	25.03 (0.38)	35.31 (1.79)	−10.28	1.2 × 10^3^***
*P. aeruginosa*	P1 (6)	21.84 (0.32)	23.53 (0.27)	−1.69	3.2 × 10^0^***
*P. aeruginosa*	P2 (7)	23.62 (0.46)	24.09 (0.23)	−0.47	1.4 × 10^0^**
*P. aeruginosa*	P3 (8)	23.56 (0.44)	23.43 (0.44)	0.13	9.1 × 10^−1^ (NS)
*P. aeruginosa*	Zymo-HostZERO (C1)	25.98 (0.23)	26.70 (0.17)	−0.72	1.6 × 10^0^***
*Staphylococcus aureus*	M-15 (this study)	20.59 (1.15)	22.49 (0.32)	−1.9	3.7 × 1^0^**
*S. aureus*	Molysis Complete 5 (C2)	22.78 (0.31)	23.33 (0.61)	−0.55	1.5 × 10^0^*
*S. aureus*	P1 (6)	21.94 (1.06)	22.74 (0.40)	−0.8	1.7 × 10^0^*
*S. aureus*	P2 (7)	22.78 (0.31)	23.46 (0.23)	−0.68	1.6 × 10^0^***
*S. aureus*	P3 (8)	22.78 (0.31)	23.04 (0.26)	−0.26	1.2 × 10^0^***
*S. aureus*	Zymo-HostZERO (C1)	26.76 (0.62)	26.07 (0.27)	0.69	6.2 × 10^−1^ (NS)

^*a*^ SD, standard deviation; Ct, cycle threshold; ΔCt, difference in quantification cycle; fold reduction was calculated as 2^ΔCt^, where each 1 Ct corresponds to a twofold change in DNA quantity. NS, not statistically significant (*P* ≥ 0.05), *** = *P* < 0.001, ** = *P* < 0.01, * = *P* < 0.05.

### Primers, probes, and amplification parameters

To assess the depletion efficiency of the protocols, universal 16S rRNA, 18S rRNA, and bacterial species-specific primers and probes were utilized (9–12). The total volume of individual qPCR reactions was 20 µL, with 2× Rotor-Gene Multiplex PCR master mix (Qiagen, Hilden, Germany) and 2 µL DNA template. qPCR reactions were performed on a Rotor-Gene Q real-time cycler for up to 40 cycles (Qiagen, Hilden, Germany). The primer/probe sequences, concentrations, and PCR cycling parameters are provided in Table S4.

### Benchmarking host depletion kits and protocols

**Fig 1:**
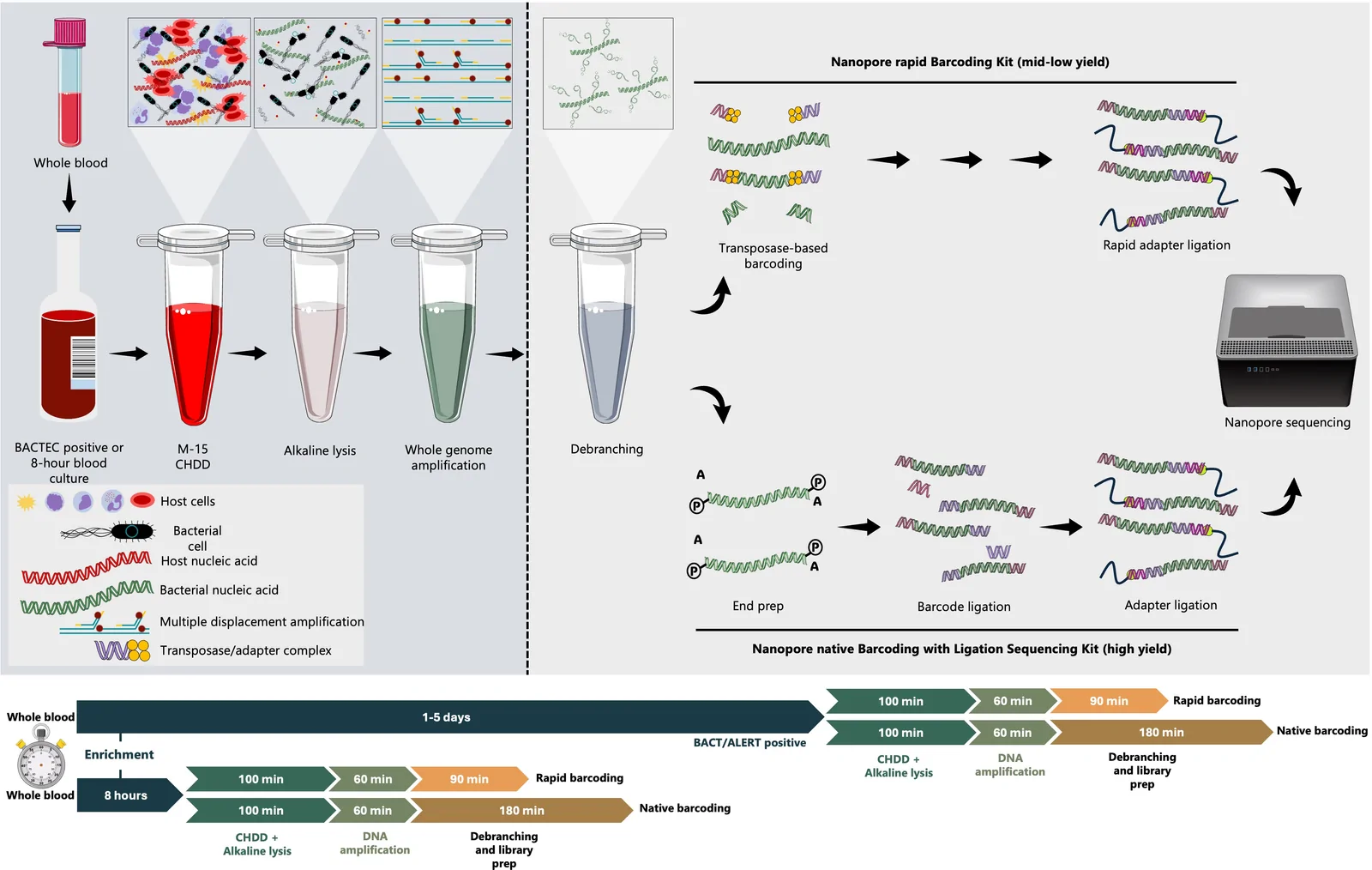
Schematic diagram depicting major steps involved in M-15 CHDD and mNGS workflow. Both BACT/ALERT flagged positive and rapid enriched (8 hour) blood culture samples can be used as input. M-15 CHDD is the first step where host DNA is selectively depleted with saponin and DNase I treatment. Host-depleted material is then subjected to alkaline lysis and whole genome amplification with REPLI-g Single Cell Kit. Amplified product is then debranched using T7 endonuclease I, and sequencing is performed with rapid barcoding (mid-low yield) or native barcoding (high-yield) kit.

A chemical host DNA depletion protocol, named M-15, was developed in this study (Fig. 1; Supplement Protocol: M-15 mNGS) and benchmarked alongside two commercial (Zymo-HostZERO [named C1; Zymo Research, CA, USA], MolYsis-Complete5 [C2; Molzym GmbH & Co, Bremen, Germany]) and three published host depletion protocols P1 (6), P2 (8), and P3 (7).

To determine host depletion efficiency and analytical sensitivity of the workflows, serially diluted (10^0^ to 10^5^ colony-forming units [CFU]/mL) exponential growth phase cells of *Escherichia coli* (ATCC 25922), *Staphylococcus aureus* (ATCC 25923), and *Pseudomonas aeruginosa* (ATCC 27853) strains were spiked in 400 µL sterile whole blood (human and sheep) and processed to deplete host cells/DNA with all six protocols. Following host depletion with P1, P2, P3, and M-15, DNA extraction was performed using QIAamp UCP Pathogen Mini Kit (Qiagen, Hilden, Germany) with the recommended homogenization step by bead beating (Pathogen Lysis Tubes L, Qiagen, Hilden, Germany). For protocols C1 and C2, DNA extraction was performed using the reagents supplied with the kits. Total DNA was extracted from the same spiked blood samples without host depletion, similarly as unenriched (non-CHDD) controls. Extracted DNA was tested using qPCR with the 16S rRNA, 18S rRNA, and species-specific primers/probes described above. Host DNA depletion was calculated from the qPCR Ct change between the depleted samples and no-host DNA depletion control. Each 1 Ct increase corresponds to approximately a twofold reduction in DNA quantity and was quantified as “Fold DNA depletion = 2^(Ct depleted – Ct control).” The statistical significance between host depletion methods (with/without chemical depletion) was evaluated using the paired Student’s *t*-test with Benjamini-Hochberg correction for multiple testing.

In addition, a subset of the simulated M-15 CHDD samples, containing 10^4^ to 10^1^ CFU/mL blood, was extracted, amplified, and barcoded for sequencing using ONT Rapid PCR Barcoding Kit (SQK-RPB004; Oxford Nanopore Technologies, Oxford, UK) and sequenced on MinION R9.4.1 flow cells. Live GPU basecalling was performed in high-accuracy mode (dna_r9.4.1_450bps_hac), and passed FASTQ reads were analyzed using Chan Zuckerberg Infectious Diseases (CZ ID) (v.0.7; https://czid.org/) Nanopore workflow (last accessed: 8 July 2024) (13).

### Determining the contribution of host depletion on bacterial cell loss/viability

Bacterial cell loss and/or viability following host depletion was determined using *E. coli, S. aureus,* and *P. aeruginosa* ATCC strains. Approximately 30 and 3 CFU of the exponential phase cells were spiked in 400 µL sterile whole blood, and the samples were processed to deplete host DNA with three top-performing protocols C1, C2, P1, and M-15. Host-depleted samples were resuspended in PBS and plated on nutrient and blood agar media to estimate bacterial concentrations using the method described by Miles and Misra (14). The same samples were plated directly without host depletion as controls. All the inoculated plates were incubated overnight at 37°C, and the number of visible colonies was recorded the next day.

### Rapid M-15 mNGS workflow tested on culture-enriched specimens

To determine the efficiency of M-15 mNGS protocol on culture-enriched samples, six culture-enriched suspected BSI samples were tested initially. Three samples flagged positive on BACT/ALERT (bioMérieux, Marcy-l'Étoile, France), and the remaining three were reported to be culture-negative. The same samples were processed directly without M-15 as no-depletion controls. To minimize hands-on time, rapid alkaline cell lysis was performed using 4 µL direct or host-depleted samples resuspended in 1 mL PBS using REPLI-g Single Cell Kit (Qiagen, Hilden, Germany). Multiple displacement amplification (MDA) was performed with the same kit for 60 minutes according to the manufacturer’s recommendation. Amplified MDA products were then debranched for 10 minutes at 37°C using T7 endonuclease (New England Biolabs, MA, United States) and subjected to 0.5× AMPure bead cleanup. Library was prepared using ONT Rapid barcoding kit (SQK-RBK004) with 400 ng amplified debranched DNA product/sample as input (Supplement Protocol: M-15 mNGS + low-medium yield protocol). The overall sample processing time of this pilot version of the workflow is approximately 4 hours (processing 6–10 samples), including 80 minutes for host depletion, 20 minutes for DNA extraction (alkaline lysis method), 60 minutes of WGA using REPLI-g Single Cell Kit, and 90 minutes for debranching, cleanup, library preparation, and flow cell loading.

To assess the efficiency of adaptive sampling (AS) with or without CHDD with M-15, rapid adapter-ligated pooled libraries (six CHDD and six no CHDD) were loaded on a MinION R9.4.1 flow cell, and the total number of channels (*n* = 512) available for sequencing were divided into two parts from the advanced options menu on the MinKNOW graphical user interface. Channels 1 to 256 were chosen for human DNA depletion using T2T-CHM13v2.0 as reference (15), and channels 257–512 for no-adaptive sampling control.

### High-yield M-15 mNGS workflow validated with culture-positive suspected BSI specimens

An additional 27 blood culture-positive (BCP) samples were tested using the optimized workflow for improved sequencing yield. Chemical host depletion, DNA extraction, MDA (60 minutes), and debranching were performed similarly to the pilot protocol. Only this time, library preparation was performed using the Ligation Sequencing Kit (SQK-LSK109) with native barcodes (EXP-NBD104) with 400 ng amplified, debranched DNA product/sample as input (Supplement Protocol: M-15 mNGS + high yield protocol). The overall sample processing time of this optimized workflow takes less than 6 hours (with 6–10 samples), including 80 minutes of CHDD, 20 minutes for DNA extraction (alkaline lysis method), 60 minutes of WGA using the REPLI-g Single Cell Kit, and about 180 minutes for debranching, library preparation, and loading.

To identify bacterial species, the CZ ID pipeline was utilized as mentioned above with a 20% abundance cutoff (200,000 base per million; BPM for CZ ID). Host-filtered contigs remaining after the 20% abundance cutoff were analyzed to identify AMR determinants with ResFinder 4.5.0 (16) using the default options. In addition, host-filtered FASTQ reads remaining after the 20% abundance cutoff were mapped against bacterial reference sequences using minimap2 (17) to estimate the breadth of coverage and sequencing depth. To determine the minimum sequencing yield required to identify bacterial species and AMR determinants, 100 megabase pairs (Mbp), 50 Mbp, 40 Mbp, 30 Mbp, 20 Mbp, and 10 Mbp raw FASTQ reads were randomly subsampled using rasusa (18) and processed similarly with CZ ID and ResFinder.

### Rapid culture enrichment to accelerate microbiological diagnosis

A shorter culture enrichment method was tested with 16 ATCC/clinical isolates representing eight gram-negative and seven gram-positive bacterial species. Approximately 1–10 CFU of actively growing (log-phase) bacterial cells were spiked in BD BACT/ALERT Standard Blood Culture media (bioMérieux, Marcy-l'Étoile, France) supplemented with 10 mL sterile whole blood (sheep), and then subjected to incubation at 36°C (± 1°C) with 150 rpm in an orbital shaker. Culture bottles were taken out at 2 hour intervals over a span of 10 hours and subsequently plated on nutrient and blood agar media using the Miles and Misra method (14). All the plates were incubated at 37°C overnight, and the number of visible colonies was recorded the next day. One milliliter of culture-enriched samples was preserved at −80°C during each sampling session to be tested later. Finally, a subset (10/16) of the 8 hour culture-enriched samples (5 gram-positive and 5 gram-negative species) was chosen and processed with the high-yield M-15 mNGS protocol (Supplement Protocol: M-15 mNGS + high yield protocol). Species, host:pathogen ratio, antimicrobial resistance genes, and minimum DNA yield per sample for identification were determined using the same pipelines and cutoffs mentioned above.

### Conventional tests for detecting species, AMR, and predicting AMR with sequencing

In the routine diagnostic microbiology laboratory, 5–10 mL EDTA-treated blood samples collected from suspected individuals were incubated in the BACT/ALERT system for up to 5 days. Samples that flagged positive were taken out and inoculated onto appropriate agars, and organisms grown were identified using MALDI-TOF mass spectrometry (Bruker Corporation, MA, USA). Phenotypic antimicrobial sensitivity was determined using disc diffusion as described by the European Committee on Antimicrobial Susceptibility Testing guideline (19) or automated systems, for example, Vitek2 (bioMérieux, Marcy-l'Étoile, France).

To compare routine phenotypic sensitivity and genotypic antimicrobial susceptibility results, AST results for the BACT/ALERT-positive BSI samples were compared to ResFinder 4.5.0 phenotype table results. Because ResFinder provides AMR predictions for 92 antimicrobial agents from 21 antibiotic classes but only predicts resistance or susceptibility (not intermediate results), only the phenotypic results common between the two (culture and sequencing) and classified as resistant or susceptible were compared. For rapid culture-enriched spiked blood samples, for which AST results were not available, whole genome sequencing (WGS) (where available) and M-15 mNGS data of the 10 ATCC/clinical isolates were compared similarly using the ResFinder 4.5.0 phenotype table. Categorical agreement, major error, and very major error were calculated using the following formula (20):


$$\;\;\;\;\;\;\;Categorical\;agreement=\frac{TR+\;TS}{Total\;number\;of\;calls\;}\times \;100$$

$$\;\;\;\;\;\;\;\;\;\;\;\;Major\;error=\frac{FR}{TS}\times \;100$$

$$\;\;\;\;\;\;\;\;\;\;\;\;Very\;major\;error=\frac{FS}{TR}\times \;100$$

where TR = true resistant, TS = true susceptible, FR = false resistant, FS = false susceptible, and where phenotypic AST is taken as the gold standard.

### Statistical analysis and visualization

All statistical analyses were performed in RStudio 2023.12.1 with R base version 4.3.3. Data cleaning, formatting, and visualization were performed using readxl, dplyr, reshape2, ggplot2, and gridExtra packages.

## RESULTS

### Chemical depletion protocols remove unwanted host with high efficiency

Protocols M-15 and P1 removed 1.5 × 10^6^ (quantification cycle ΔCt −20.52) and 1.8 × 10^5^-fold (ΔCt −17.45) host DNA, respectively, from the whole blood samples spiked with *Escherichia coli*, *Staphylococcus aureus*, or *Pseudomonas aeruginosa* ATCC strains, with depletion efficiency over 99.99%. Commercial kit C1 removed 99.82% (6.9 × 10^2^-fold) host, and C2, 97.93% (3.7 × 10^1^-fold). Approximately 81.09% and 59.19% host were removed with the protocols P2 and P3, respectively (Table 1). On average, 5.58% (P3), 25.96% (C1), 33.09% (P2), 39.86% (P1), 42.77% (C2), and 51.63% (M-15) bacterial DNA were removed during host depletion with the six kits/protocols (Table 1). Protocols with higher depletion efficiencies also exhibited increased bacterial DNA loss (Table 1).

Protocol C2 exhibited a strong negative bias towards *P. aeruginosa*, as >99% (ΔCt −10.2) loss was observed, possibly due to the detrimental effect of buffer CM (a selective host cell lysis buffer provided in the kit) on this bacterial species during the host lysis steps. In a recent study, where blood culture-positive samples were processed with protocol C2 to deplete host and enable rapid sequencing-based species and AMR identification (20), sequencing results also indicated suboptimal accuracy of C2 mNGS for *P. aeruginosa* compared to other bacterial species, possibly indicating similar lysis bias.

### CHDD protocols perform poorly with samples containing low bacterial concentrations

**Fig 2:**
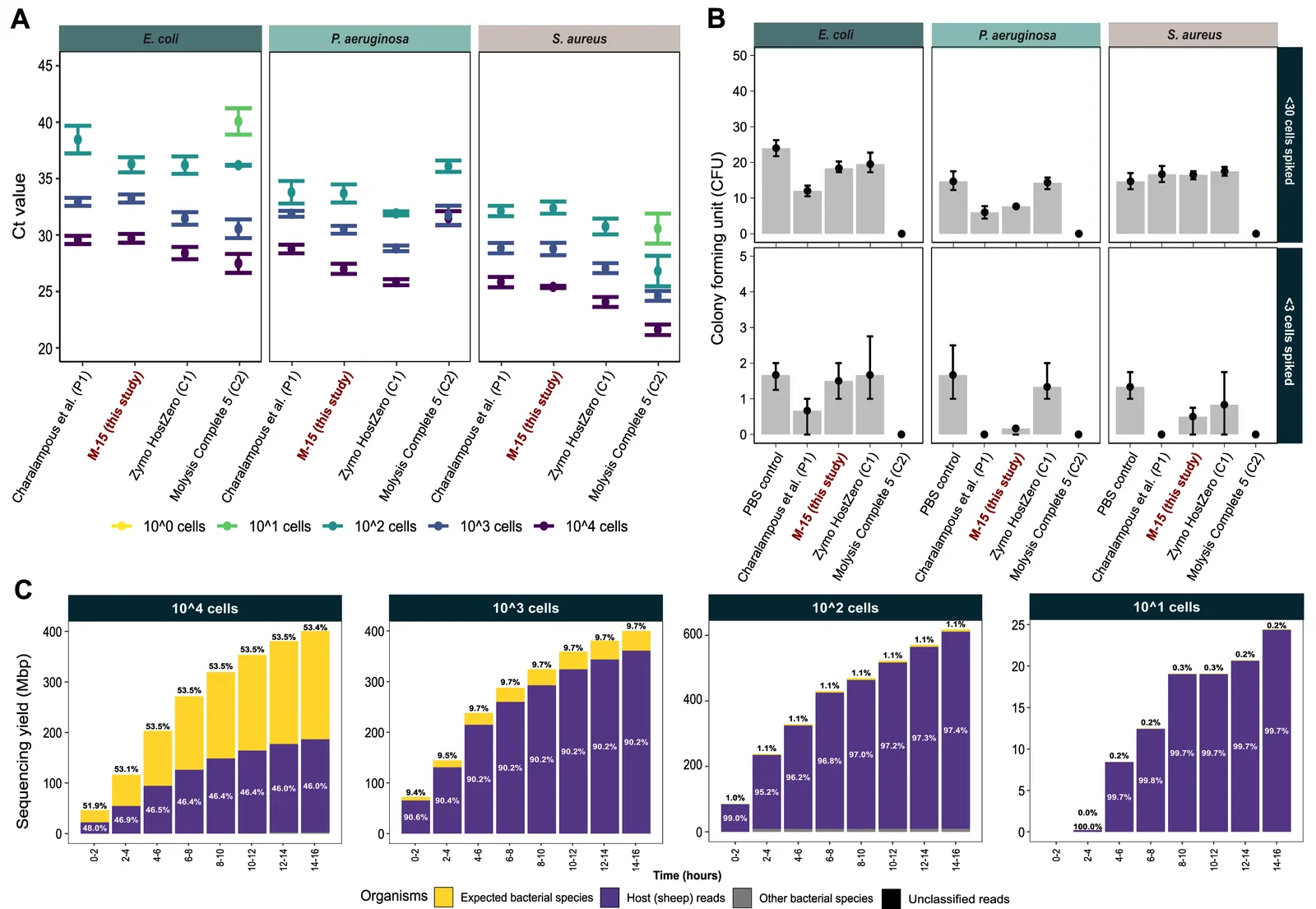
Overall performance of M-15 versus published/commercial protocols. (**A**) qPCR Ct values (median and standard deviation) of different concentrations (10^4^ to 10°CFU/mL; see legend) of three ATCC strains (*Escherichia coli*, *Pseudomonas aeruginosa*, and *Staphylococcus aureus*) spiked in whole blood and processed with top CHDD kits/protocols (P1, M-15, C1, and C2) performing the best in terms of host DNA depletion. (**B**) Number of viable cells recovered from blood samples spiked with <30 and <3 CFU of the three ATCC strains following CHDD with P1, M-15, C1, and C2, compared to the no-CHDD/PBS control. (**C**) Proportion of expected bacteria initially spiked in blood for M-15 CHDD, compared to host (sheep) DNA, other bacterial species (different from the three species spiked), and unclassified reads, over 16 hours of MinION sequencing. The four facets are showing simulated/spiked bacterial concentration ranging from 10^4^ to 10^1^ CFU/mL of sheep blood.

Although the host depletion efficiency of some of the protocols, including M-15, was encouraging, we observed some bacterial loss. Therefore, we aimed to evaluate their applicability on blood samples with low bacterial concentrations (1 to 100 CFU/mL), as typically seen in BSI cases (21). For this, we selected the top-performing protocols, M-15, P1, C1, and C2, which exhibited the highest host depletion efficiency. For all three ATCC strains tested (*E. coli, S. aureus, P. aeruginosa*), qPCR analytical sensitivity of protocols M-15 (mean Ct 34.93 ± 1.98), P1 (mean Ct 33.23 ± 2.21), and C1 (mean Ct 32.36 ± 2.39) was 10^2^ CFU/mL. With protocol C2, qPCR was able to detect up to 10^1^ CFU/mL *E. coli* and *S. aureus* (mean Ct 33.79 ± 5.08); however, like before, Ct values from *P. aeruginosa*-spiked CHDD samples were consistently very high (Ct >30) across all five *P. aeruginosa* concentrations (10^0^ to 10^4^ CFU/mL) spiked in whole blood (Fig. 2A).

To understand which part (chemical wash or DNA extraction) of the CHDD protocols is contributing to reduced recovery of bacterial DNA, we processed low abundant spiked samples (<30 and <3 CFU/mL) with M-15, P1, C1, and C2 protocols and confirmed viability/recovery with culture. Viable plate counts following CHDD showed protocols C1 retained 92%, M-15 76%, and P1 62% of bacteria of the low initial counts of <30 cells spiked in blood (Fig. 2B). With even lower concentration (<3 cells), the overall bacterial recovery rates with protocols C1, M-15, and P1 were 72%, 43% and 13%, respectively, compared to the no-CHDD controls (Fig. 2B). No viable bacteria were recovered using protocol C2 for any of the bacterial species or samples tested; again, possibly indicating buffer CM’s effect on their growth or viability, although *P. aeruginosa* was the only species (specifically ATCC 27853) tested in this study that displayed suboptimal performance with downstream molecular assays (Fig. 2B).

In order to estimate the proportions of expected bacterial DNA (i.e., bacterial species spiked initially) versus host and others (unclassified and other species), a subset of simulated M-15 CHDD samples (highest host depletion efficiency; 4.1 × 10^6^-fold), spiked with 10^4^ to 10^1^ *E. coli* CFU/mL, were sequenced. While the host-to-bacteria DNA ratio might differ between bacterial species due to the difference in their genome size, this would facilitate estimating the lowest bacterial concentration (CFU/mL) that would most likely provide consistent results with downstream molecular applications, especially sequencing. The proportion of expected bacterial (*E. coli* that were initially spiked) DNA sequence yield Mbp) in M-15 CHDD samples was 53.4% and 9.7% of total DNA for samples containing 10^4^ and 10^3^ CFU/mL bacteria, respectively. For low abundant samples, bacteria represented only 1.1% (10^2^ CFU/mL) and 0.2% (10^1^ CFU/mL) of total DNA yield following >99.99% host removal with M-15 (Fig. 2C). Proportion of DNA sequence yield from expected bacterial species compared to the host remained unchanged throughout the sequencing run regardless of bacterial concentration spiked or DNA ratio (Fig. 2C). The suboptimal host-to-pathogen ratio in low abundant blood samples meant that sequencing-based assays may not yet provide satisfactory results even with CHDD, unless an additional enrichment and/or depletion step is included pre- and/or post-DNA extraction to further improve bacterial proportions, and a more robust and sensitive DNA extraction method is benchmarked and validated.

### M-15 mNGS and adaptive sampling significantly improve bacterial proportions

Due to suboptimal performance of M-15 mNGS observed with low bacterial concentrations directly in blood samples, three BACT/ALERT-positive (BCP01-BCP03) and three negative (BCN01-BCN03) culture-enriched samples from suspected BSI cases were initially tested with a rapid version of M-15 CHDD protocol (Fig. 1).

**Fig 3:**
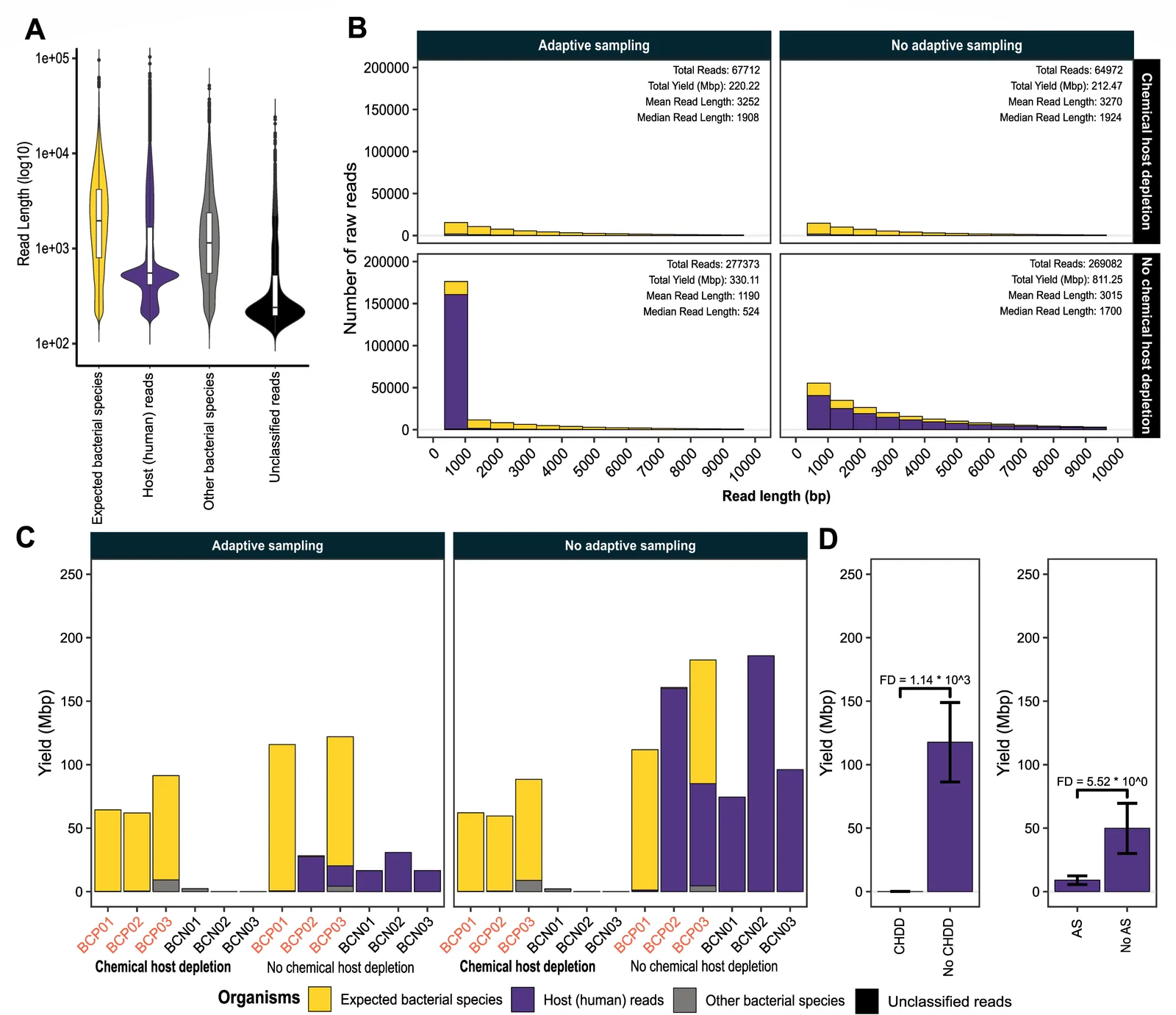
Host depletion efficiency of M-15 mNGS and AS, versus no CHDD and no AS, tested with patient samples. (**A**) Violin plot showing size distribution of sequencing reads aligned to expected bacterial species, host (human), other bacterial species, and reads for which there were no matches within the BLAST Nr/Nt database. (**B**) Histogram highlighting the number of raw sequencing reads from expected bacteria, host, others (other bacteria or unclassified). Colored facets subdividing the raw reads further to distinguish read length variation by AS, no AS, CHDD, and no CHDD. (**C**) Overall sequencing yield of CHDD and no-CHDD samples sequenced, comparing AS and no-AS. Stacked colored bars highlight the proportion of DNA aligned to host, bacteria, and others/unclassified species (see legend). BCP samples are shown in red text, while blood culture-negative (BCN) samples are shown in black. (**D**) Depletion efficiency (fold difference, FD) in DNA yield (Mbp) of sequences from host with CHDD and AS compared to no-CHDD and no-AS.

Length of the sequencing reads varied significantly among organisms, with expected bacterial species (species that were identified with culture-based methods) having the highest median read length (1,957 bp); this was 8.15× longer than the unclassified reads (240 bp), 3.52× longer than human reads (552 bp), and 1.7× longer than other bacterial reads (mixed bacterial species not expected from the sample; 1,145 bp) (Fig. 3A). The read length difference between host and bacteria could be the result of CHDD, as host DNA was digested with DNase I following selective lysis, meaning a stringent SPRI bead washing step could possibly reduce the unwanted host background even further.

Overall yield in the AS channels (1–256) was 1.86× lower (550.33 Mbp AS vs 1,023.72 Mbp no-AS) than no-AS (channels 257–512), although the total number of reads for AS and no-AS samples was almost the same, with 345,085 and 334,054 reads, respectively. Median read length for AS channels was 3.2× shorter than the no-AS, and host constituted >90% of the reads that were smaller than 1 Kb in AS channels (Fig. 3B). This illustrates that AS influenced the length of sequenced fragments as a result of its limited ability to deplete shorter host reads (≤1 Kbp), which were highly abundant in the directly extracted no-CHDD samples.

Samples processed with M-15 CHDD had 1.14 × 10^3^ times less human DNA compared to the no-CHDD controls (based on sequencing yield in Mbp; Fig. 3D). Samples sequenced using AS had 5.52-fold less human DNA compared to the controls; however, no bacterial enrichment was observed (Fig. 3C and D). This highlights the importance of host depletion even in positively flagged culture-enriched specimens, as it may lead to lower sequencing costs per sample, and more importantly, reduce the sequencing time required to reach a desired yield for determining the bacterial species responsible for the BSI.

### M-15 mNGS accurately predicts mono-bacterial species in BACT/ALERT-positive samples

Because suboptimal sequencing yield was observed when debranched M-15 CHDD MDA samples were initially processed (rapid M-15) using the rapid barcoding kit (Fig. 1), it was decided to utilize the Ligation Sequencing Kit with Native Barcodes (Fig. 1). An additional 27 culture (BACT/ALERT) positive BSI blood samples were processed with the high-yield M-15 mNGS protocol.

**Fig 4:**
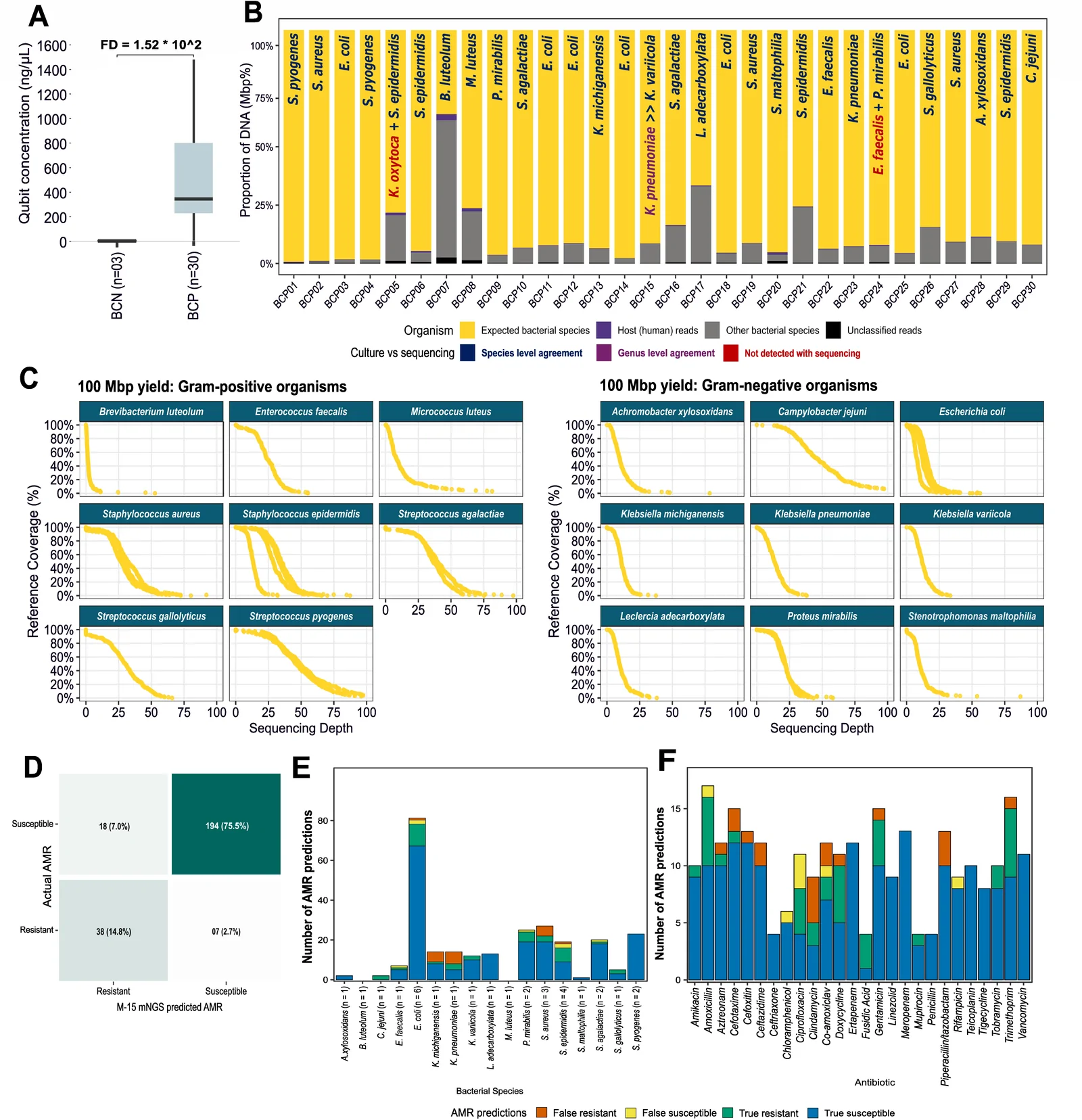
Species and antimicrobial resistance prediction accuracy of M-15 mNGS. (**A**) DNA concentration (ng/μL) of three blood culture-negative (BCN01–BCN03) and 30 blood culture-flagged positive (BCP) samples, following M-15 CHDD, and whole genome amplification with the REPLI-g Kit. Quantification was performed with the Qubit broad range dsDNA assay. (**B**) Proportion of expected bacterial DNA (yellow bar chart; confirmed by culture) versus all other reads following M-15 CHDD and MinION sequencing with native barcoding kit. BACT/ALERT-positive samples that have species-level agreement with culture have text labeled dark blue, those with genus-level agreement are purple, and those not detected by sequencing are labeled red. (**C**) Cumulative depth for eight gram-positive and nine gram-negative organisms from 30 BACT/ALERT-positive blood culture samples following M-15 mNGS, with 100 Mbp sequencing yield per sample. (**D**) Number and proportion of true positive, true negative, false positive, and false negative AMR predictions with M-15 mNGS compared to the standard culture-based AMR prediction for 30 BACT/ALERT-positive blood culture samples. (**E**) Number and proportion of ResFinder true positive, true negative, false positive, and false negative AMR predictions with M-15 mNGS by bacterial species from the 30 BACT/ALERT-positive blood culture samples tested in this study. (**F**) Number and proportion of true/false AMR predictions with mNGS for the same culture-positive samples categorized by antibiotics tested. Prediction of AMR profile with mNGS was done only using the resistance and susceptibility results from antibiotics that were present in both phenotypic and genotypic data sets (*n* = 257).

Overall, BCP samples subjected to CHDD had 152.4× higher DNA concentration compared to culture-negative (BCN) samples (BCP CHDD 509.02 ng/µL versus BCN CHDD 3.34 ng/µL, as measured using Qubit) after 60 minutes MDA (Fig. 4A; Table S1). However, directly extracted and amplified blood culture-negative samples yielded 753 ng/µL DNA on average following 1 hour MDA with REPLI-g Kit (Table S1). Four bacterial species with high GC content, *Brevibacterium luteocum* (GC 67.8%), *Micrococcus luteus* (GC 74.0%), *Stenotrophomonas maltophilia* (GC 66.7%), and *Achromobacter xylosoxidans* (GC 64.0%) yielded low DNA (15.7× less than other species), with an average of 32.23 ng/µL after 1 hour MDA; however, this was still 9.64× higher than BCN samples following CHDD (Table S1). The potential reason behind low yield in species with high GC% could involve stable secondary structure or denaturation difficulties, as highlighted in some studies (22, 23).

After testing multiple abundance cutoffs with CZ ID, a 20% abundance (200,000 BPM) cutoff was selected as it provided the best overall sensitivity (93.75%) and specificity (100%) for species identification with M-15 mNGS. Using this cutoff, sequencing correctly identified 28/28 mono-bacterial species, 3/3 negative samples, and 2/4 multi-bacterial species found in two samples (Fig. 4B; Table S5).

Initially, it was assumed that the two species (*Klebsiella oxytoca* and *Enterococcus faecalis*) in two mixed samples (BCP05 and BCP24) were missed due to bacterial cell lysis during CHDD (Fig. 4B). However, some of the mono-bacterial samples tested in this study contained the same bacterial genera/species, and no negative bias was observed with sequencing following CHDD. Also, all the CHDD-flagged positive samples (*n* = 30, species = 17) were cultured overnight to confirm viability, and only one (*M. luteus*) showed visible growth impairment on agar plates (Table S1). Therefore, it is likely that these two organisms were missed with sequencing due to their relatively low abundance compared to the other species present in the sample. For one sample (BCP15), *Klebsiella pneumoniae* was identified with culture but *Klebsiella variicola* with mNGS; and the species was later confirmed to be *K. variicola* with 16S rRNA amplicon sequencing. Overall, expected bacterial species constituted 88.7% of total sequencing yield versus human, which represented only 0.46% of total DNA (Fig. 4B).

To understand the breadth of coverage across the bacterial genomes with 100 Mbp sequencing yield per CHDD sample, 100 Mbp FASTQ reads subsampled with rasusa were analyzed. For every 100 Mbp DNA sequenced per sample, 96.76% and 99.48% coverage could be achieved with at least 1× depth for the gram-positive and gram-negative organisms tested, respectively. For 5× and 10× depth, gram-positive organisms had a mean genome coverage of 87.89% and 78.29% and gram-negatives 95.28% and 78.38%, respectively (Fig. 4C). For all the 30 blood culture-positive samples tested (BCP01 to BCP30), >20% abundance cutoff accurately identified the present bacterial species with as low as 10 Mbp sequencing yield per sample (Table S5).

### Rapid culture enrichment can facilitate same-day species/AMR identification

When a blood culture is flagged as positive, it contains approximately 10^7^ to 10^9^ bacterial cells/mL of culture broth (24), which is more than the input concentration (≥10^4^ CFU/mL) required for the M-15 mNGS to work reliably, as observed during our initial experiments (Fig. 2C). This meant that a shorter culture enrichment step could further accelerate the microbiological diagnosis, and M-15 mNGS could be utilized even before a sample is flagged positive. However, given the variety of bacterial species involved in BSI and differences in their growth rate, we aimed to understand the minimum incubation period required to reach that threshold with a representative range of clinically important bacterial species (*n* = 15).

**Fig 5:**
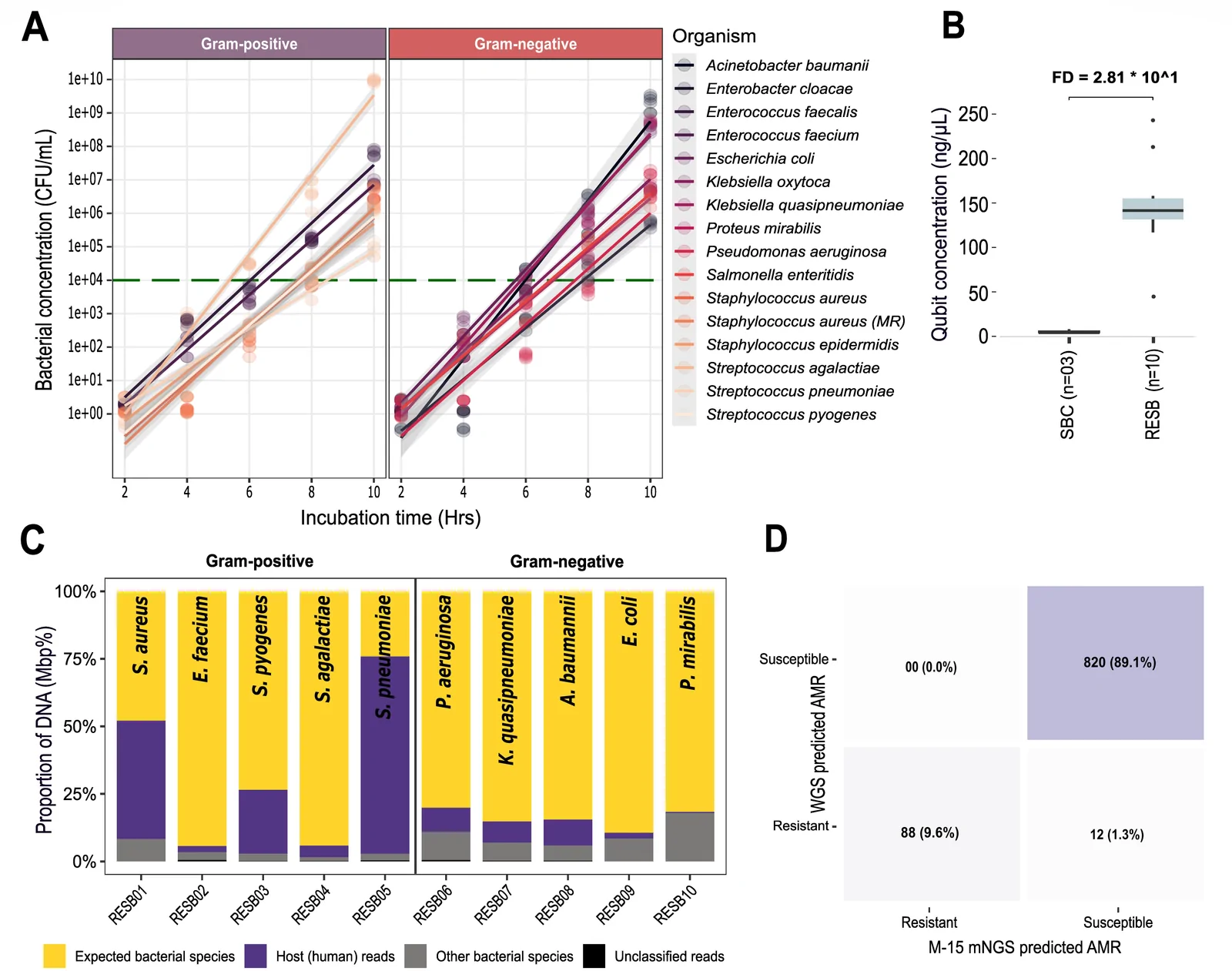
Rapid culture enrichment: species covered, overall accuracy in species and antimicrobial resistance prediction. (**A**) Time to reach 10^4^ CFU/mL (green line; optimal concentration for CHDD with culture-based bacterial enrichment prior to M-15 CHDD and mNGS, tested with the top 15 bacterial species causing bloodstream infection. (**B**) DNA concentration (ng/μL) of the three sterile culture-enriched blood (SBC01–SBC03) and 10 rapid (8 hours) culture-enriched spiked blood samples, following M-15 CHDD, and whole genome amplification with the REPLI-g Kit. Quantification was performed with Qubit broad range assay. (**C**) Proportion of expected bacteria (yellow; spiked initially) versus all other reads following M-15 CHDD and MinION sequencing. Species, genus, and non-agreements from rapid culture-enriched samples are highlighted with the same colored labels as before. (**D**) Number and proportion of ResFinder true positive, true negative, false positive, and false negative AMR predictions with M-15 mNGS compared to WGS results of the same isolates/strains. MR, methicillin-resistant.

Four of 15 bacterial species tested (*Streptococcus agalactiae, Acinetobacter baumannii, Klebsiella pneumoniae,* and *Escherichia coli*) reached 10^4^ CFU/mL, the optimal concentration for M-15 mNGS, under 6 hours of culture enrichment (Fig. 5A). With 8 hour incubation, 12/15 species reached the desired threshold of 10^4^ CFU/mL. The remaining three species, *S. aureus, Streptococcus pneumoniae,* and *P. aeruginosa*, were slightly lagging, reaching approximately 6.04 × 10^3^ CFU/mL on average with 8 hour enrichment. All the species (15/15) tested reached/crossed the threshold under 10 hours of culture enrichment (Fig. 5A).

To identify proportions of expected bacteria obtained using M-15 mNGS after 8 hour rapid culture enrichment, three sterile blood culture samples and a subset of 10 bacterial species (5 gram-positive and 5 gram-negative, including the three slow-growing species tested earlier) were selected and processed with this approach (Fig. 5A and C). One potential costly drawback with the rapid enrichment approach could be the necessity to blindly sequence all blood cultures if the positive and negative samples could not be differentiated prior to sequencing. However, following CHDD and 60 minutes of MDA, the rapid culture-enriched spiked blood samples (*n* = 10) showed 28.16× higher dsDNA concentration (154.6 versus 5.49 ng/µL) on average compared to sterile human blood enriched as negative controls (SBC, *n* = 3), providing a unique signature for the detection of positive samples prior to sequencing (Fig. 5B; Table S2).

Following sequencing, expected bacteria represented 89.4% of total DNA yield on average for seven samples that reached 10^4^ CFU/mL under 8 hours versus 50.06% for the remaining three samples having relatively slow-growing species. All samples combined, 75.4% of the total DNA yield matched with the bacterial species initially used for spiking and enrichment and 17.64% with host species (Fig. 5C). Similar to BACT/ALERT-positive samples, >20% abundance threshold successfully identified 10/10 bacterial species sequenced to validate the rapid enrichment protocol (Fig. 5C). Also, like before, as low as 10 Mbp yield (subsampled) was sufficient to accurately identify bacterial species with the abovementioned cutoff (Table S6).

### *In silico* prediction of AMR is promising but requires improvement for clinical use

The accuracy of AMR prediction for the BACT/ALERT-positive samples was determined by comparing the ResFinder phenotype antibiotic data with the phenotypic culture-based AST results (*n* = 257). For all 30 culture-positive samples, mNGS exhibited 90.27% (232/257) categorical agreement overall, with 9.28% (18/194) major error and 18.42% (7/38) very major error (Fig. 4D). Among the 17 bacterial species, discrepancies in resistance calls for *Klebsiella michiganensis* (5/18), *K. pneumoniae* (6/18), and *S. aureus* (5/18) contributed to the highest number of major errors, and *E. coli* (2/7) and *Staphylococcus epidermidis* (2/7) to very major error (Fig. 4E). Of 27 antibiotics for which results were compared, very major error for ciprofloxacin was most common (3/7), possibly due to the relatively low accuracy of ONT reads and the frequent association of resistance to fluoroquinolones with mutations in quinolone resistance-determining region of DNA gyrase and topoisomerase-encoding genes, as opposed to resistance encoding gene presence/absence in other antibiotics (25). Clindamycin (4/18) and piperacillin/tazobactam (3/18) were the top 2 antibiotics exhibiting major errors (Fig. 4F).

Due to the smaller number of ATCC strains and clinical isolates (*n* = 10) tested, as well as unavailability of phenotypic AST data for a few clinical isolates, AMR predictions of M-15 mNGS with the rapid culture method were compared to WGS of pure bacterial strains predicted AMR of the same strains/species using ResFinder (*n* = 920 predictions) (Table S3). Overall categorical agreement for M-15 mNGS versus WGS was 98.7% (908/920) and mNGS exhibited 89.1% true susceptible, 9.6% true resistant, 1.3% false susceptible, and no false resistant predictions (Fig. 5D). *P. aeruginosa* was the only species for which sequencing results did not match perfectly with its reference genome (12/92 predictions), and therefore was the only source of false susceptibility, possibly due to its high GC content leading to inefficient/uneven DNA amplification with the REPLI-g Kit (Table S2).

For both BACT/ALERT-positive blood culture and rapid enriched spiked blood M-15 CHDD blood, ≥50 Mbp sequencing yield was sufficient to predict AMR profiles, with the accuracy almost the same as having a sequencing yield of >100 Mbp per sample (Tables S7 and S8).

## DISCUSSION

Timely identification of pathogens and their antimicrobial resistance profile is crucial in BSI because more targeted and effective antibiotic therapy can minimize the progression and severity of the disease and therefore lead to improved patient outcomes. Rapid sequencing-based methods, especially in combination with selective chemical depletion approaches, have the potential to reduce turnaround time and therefore lessen detrimental outcomes. In working toward this goal, this study initially assessed the effectiveness of five published and commercial, one in-house, and one computation-based host depletion strategies.

As a first step, we compared the effectiveness of an in-house host depletion method (M-15) with five published and commercial approaches, as well as a computation-based host depletion strategy. The host depletion efficiency of most of the CHDD workflows was outstanding (particularly for M-15 and P1). However, we also noticed detrimental effects of some selective host lysis chemicals (e.g., buffer CM) toward a few clinically important bacterial species. Some previous studies have also highlighted potential negative biases introduced by differential lysis methods, meaning the selection of chemicals for selective host depletion is crucial (26). In this study, we found that 2.25% saponin used for CHDD had minimal effect on viability, as only *Brevibacterium luteolum* and *S. pneumoniae*, 2 out of 22 clinically important bacterial species tested, were non-viable following saponin treatment (Tables S1 and S2). For samples with low bacterial abundance, the proportion of bacterial DNA was insignificant, ≤1% of total DNA, even after M-15 CHDD, which can remove >99.99% host DNA. However, bacteria represented >50% of total DNA sequence yield for the CHDD samples containing ≥10^4^ CFU/mL bacteria. This meant additional enrichment/depletion was necessary to get improved proportions of the sequencing yield that is bacterial. As a simple and cost-effective alternative, we realized that a culture-based enrichment step prior to CHDD and sequencing might improve DNA extraction efficiency/robustness and proportion of bacteria in the sample simultaneously.

Therefore, we initially tested BACT/ALERT-positive samples with two versions of M-15 mNGS protocols. As confirmed during the initial phase, the proportion of host-to-bacterial DNA sequenced was improved significantly with CHDD and AS. However, the sequencing yield was suboptimal when CHDD whole genome amplification (WGA) was paired directly with the Rapid Barcoding Kit (not systematically tested). The best output was observed when debranched MDA products were barcoded using the Ligation Sequencing Kit, although it requires additional hands-on time and potentially pricier add-on reagents for library preparation (e.g., NEBNext Companion Module for Oxford Nanopore Technologies). Both versions of the workflows have their own strengths and limitations; for example, the rapid workflow requires less hands-on time but may result in relatively low sequencing yield, leading to higher sequencing cost per sample (Fig. 1). In contrast, the high-yield workflow would reduce sequencing cost (approximately £60/sample) by providing greater output with only 90 additional minutes for sample processing (Fig. 1). Several studies have already highlighted this difference (27, 28); consequently, users may choose the workflow that best suits their need/budget.

Although there are multiple bioinformatic pipelines available for mNGS-based species identification, only a few have been developed and standardized for diagnosing bloodstream infection. The aim of this study was therefore not to benchmark these pipelines; instead, we assessed the utility of one of the well-regarded workflows, CZ ID, in combination with M-15 mNGS. With the 20% (200,000 BPM) abundance cutoff, sequencing accurately identified 3/3 negative, 28/28 mono-bacterial, and 2/4 multi-bacterial species reported in two samples, with >88% DNA classified as the expected species. For the two polymicrobial samples (BCP05 and BCP24), one of two bacterial species was detected in each case. This is possibly because of the low abundance of the two species, *Klebsiella oxytoca* and *Enterococcus faecalis,* that were missed. Several other sequencing-based studies have highlighted this issue (20), and further research is required to increase the overall sensitivity of M-15 mNGS or sequencing in general to identify multi-bacterial infections.

To determine minimum sequencing yield required to accurately identify bacterial species, we randomly subsampled the reads under the established assumption (Fig. 2C) that the proportion of host-to-bacteria remains consistent throughout a sequencing run (29). The results demonstrated that the species prediction accuracy with the given cutoff remains nearly identical even with 10 Mbp yield per sample. Because the primary aim of this study was rapid species and AMR identification, it is not entirely necessary to generate hundreds of Mbp reads/sample to cover almost the entire genome with high sequencing depth for patient management unless the aim is to utilize these data for hospital outbreak investigation. However, considering the variability in sequencing depth and host background, the exact cutoff should be determined site-wise with more positive/negative clinical samples.

With the rapid culture enrichment, only a few species tested reached/crossed ≥10^4^ CFU/mL threshold under 6 hours, and at least 8 hours of incubation was required to get more comprehensive representation of critically important gram-positive (*n* = 7) and gram-negative (*n* = 8) bacterial pathogens. M-15 CHDD and sequencing of a subset of rapid (8 hour) enriched samples (5 gram-positive and 5 gram-negative) further confirmed our hypothesis: sequence reads from expected bacteria represented >75% of total yield, and similar to BACT/ALERT-positive samples, only 10 Mbp reads accurately identified species with the previously assigned abundance threshold.

Regarding AMR prediction, it is worth mentioning that not all expected molecular mechanisms of antibiotic resistance have been determined (30). Additionally, a mere presence or absence of genes and/or mutations does not always correlate to phenotypic resistance or susceptibility (30). Therefore, at present, it is less likely that a sequencing-based approach would match culture-based phenotypic results with 100% accuracy for all the bacterial species commonly identified in BSI. Like species identification, several workflows can predict AMR using mNGS data. The underlying principles, databases, and/or accuracy can vary among these workflows for AMR identification (31). Therefore, it was decided to test ResFinder, a well-regarded workflow, with M-15 mNGS, as benchmarking several workflows/bioinformatic pipelines was not within the scope of this study. M-15 mNGS combined with ResFinder exhibited overall 90.3% accuracy, 9.28% major, and 18.42% very major error with BACT/ALERT positive samples. These proportions closely match with an earlier study that utilized culture-enriched samples for metagenomic sequencing (20).

With rapid culture-enriched samples, WGS data were used as a comparator. Although this is less ideal than comparing with culture-based phenotypic results, we wanted to understand how closely mNGS results matched those of reference sequences of bacteria that were used for spiking. With 920 ResFinder AMR predictions, mNGS showed 98.7% categorical agreement, and only 1.3% false susceptibility, primarily due to *P. aeruginosa*, the only species exhibiting discordant results. Similar suboptimal results with *P. aeruginosa* were also observed in the aforementioned study (20). However, in our case, this likely stemmed from inefficient/uneven DNA amplification due to high GC content of *P. aeruginosa* (66.6%), as we did not observe any lysis/viability issues with culture when CHDD samples were plated on agars (Table S2). As an alternative to REPLI-g, more robust whole genome amplification methods can be explored in the future to minimize the GC content-based amplification bias we observed in this study.

It is important to recognize that this study had several limitations. Since we only had access to different volumes of surplus EDTA-treated blood samples from the suspected BSI cases, stored in a refrigerator for up to 5 days until reporting was complete, it was decided not to test the rapid enrichment workflow on patient samples. Instead, 15 clinically important bacterial species were chosen for initial validation. This is because the different volume and extended storage of the patient samples might affect viability, and/or change the actual growth of the bacteria and therefore might not be an appropriate representative of freshly drawn samples. However, to fully validate M-15 mNGS, it is necessary to test the rapid enrichment approach using a large cohort of freshly collected positive and negative blood samples in a clinical setting. It is also worth mentioning that this rapid 8-hour enrichment period may fall within a window where some commercially available molecular diagnostic platforms can conveniently provide actionable results without metagenomic sequencing. However, compared to many of these targeted panels, M-15 mNGS can still offer additional benefits in cases where higher resolution genomic information obtained with higher sequencing yield can guide Infection Prevention and Control measures by providing strain-level data.

Also, the rapid and high-yield mNGS workflows were benchmarked, and a few batches of samples were initially processed using R9.4.1 flow cells, before more accurate R10.4.1 kits became widely available on the market. Therefore, it was decided to continue testing all the samples with library kits compatible with R9.4.1 flow cells (27). We believe that the performance of M-15 CHDD would be further enhanced with the use of newer versions of kits/flow cells, as a higher proportion of ≥Q20 reads would likely allow mutations associated with AMR (e.g., *gyrA/parC*) to be more reliably predicted.

Considering the overall performance, it seems more work is required in terms of wet/dry lab to make AMR predictions more accurate to be accepted as a replacement of blood culture and phenotypic assays. This is important especially in cases of very major errors observed in this study as they might lead to suboptimal antibiotic selection. Inclusion of multiple AMR databases/tools (e.g., CARD, ARIBA) to create a hybrid pipeline and/or utilizing machine learning models for phenotypic AMR prediction may yield more accurate results and development of such tools for M-15 mNGS could be pursued in the future (32, 33). However, as an addition to existing culture-based methods, M-15 mNGS at its current stage still holds promise to inform treatment in comparison to empirical antibiotic therapy. For clinical management of BSI or infection in general, the accuracy of empirical treatment can range from 20% to 80% (34–36). This means that a high proportion of cases receive inappropriate antibiotics until more targeted therapy is administered utilizing the phenotypic AST results (conventionally available in 48–72 hours), potentially risking detrimental outcomes for critically ill patients (35). With the rapid availability of bacterial species and AMR predictions that surpass empiric prescription and therapy, mNGS can significantly refine the choice of antimicrobials, increasing the likelihood of favorable outcomes. However, because the confidence of phenotypic AMR prediction using genome data can be affected by many factors, a thorough clinical validation would be required and a hybrid method (combining search algorithms), AMR database, or machine learning-based approach could be implemented for rapid M-15 mNGS to be recommended for clinical implementation.

As sample processing cost with M-15 mNGS is roughly around £120 to £160 per sample (depending on library kit, flow cells, and the number of samples multiplexed), a possible costly drawback especially when implementing the rapid enrichment approach was the necessity to blindly sequence all blood cultures if the culture-positive and negative samples could not be differentiated prior to sequencing. However, when compared to the negative blood samples, both BACT/ALERT-positive and rapid enriched spiked M-15 CHDD blood had 152× and 28× higher dsDNA concentration following MDA, meaning any negative samples could potentially be filtered out prior to sequencing if their Qubit-measured DNA concentration provides a differentiable signature, as we observed following CHDD and WGA (Tables S1 and S2). However, the exact cutoff must be determined on-site (different clinical settings or in different laboratories) with more negative patient samples before implementation in a clinical setting. It is important to note that this differentiation is only possible with rapid CHDD, as direct extraction and amplification would not provide a discernible characteristic for negative samples due to the overwhelming abundance of host DNA masking the unique signature from the bacteria (Table S2). This approach may miss the identification of some slow-growing and/or bacterial species with high GC% as negative. However, because M-15 mNGS is currently intended to complement existing technologies rather than replace them, species that might be missed would still be captured with traditional culture-based methods.

Overall, this study demonstrates that, with careful optimization, CHDD and metagenomic sequencing approaches such as M-15 mNGS can meaningfully complement existing diagnostic tools for bloodstream infections. By improving the speed and accuracy of pathogen and AMR identification, these methods offer the potential to refine empirical treatment decisions and ultimately improve patient outcomes in clinical practice. As sequencing, data analysis, and depletion/enrichment technologies such as M-15 mNGS continue to evolve, the existing limitations or challenges are likely to diminish over time. The journey toward that goal may be difficult but is worth pursuing considering the potential of these new and innovative technologies in transforming healthcare and turning the tide in the fight against AMR and infectious diseases.

## Supplementary Material

Reviewer comments

## Data Availability

All the raw sequencing reads (fastq.gz: host excluded) analysed and presented in this study are available on European Nucleotide Archive under the study accession “PRJEB80726”. Data set link: https://www.ebi.ac.uk/ena/browser/view/PRJEB80726.

## SUPPLEMENTAL MATERIAL

Supplemental tables and supplemental protocols spectrum.03240-25-s0001.docx Tables S1 and S2: DNA yield/viability tables for clinical and 8-h culture-enriched samples. Tables S5 to S8: Species ID and AMR detection performance across sequencing depths. Tables S3: AMR concordance for selected isolates. Tables S4: Primer/probe lists. Supplemental protocols: The full M-15 CHDD + WGA + library prep protocol.

OPEN PEER REVIEW reviewer-comments.pdf An accounting of the reviewer comments and feedback.

ASM does not own the copyrights to Supplemental Material that may be linked to, or accessed through, an article. The authors have granted ASM a non-exclusive, world-wide license to publish the Supplemental Material files. Please contact the corresponding author directly for reuse.
