# Supplementary material for: Rapid and modular workflows for same-day sequencing-based detection of bloodstream infections and antimicrobial resistance determinants using culture-enriched samples
Source: Microbiol Spectr. 2026 Jun 15;14(7):e03240-25. doi: 10.1128/spectrum.03240-25 (PMC13339894; doi:10.1128/spectrum.03240-25)
Supplement: Supplemental tables and supplemental protocols — Tables S1 and S2: DNA yield/viability tables for clinical and 8-h culture-enriched samples. Tables S5 to S8: Species ID and AMR detection performance across sequencing depths. Tables S3: AMR concordance for selected isolates. Tables S4: Primer/probe lists. Supplemental protocols: The full M-15 CHDD + WGA + library prep protocol. [file spectrum.03240-25-s0001.docx]

**Title:**

Rapid and modular workflows for same-day sequencing-based detection of bloodstream infections and antimicrobial resistance determinants using culture-enriched samples

**Authors:**

Mohammad Saiful Islam Sajib^1^*, Katarina Oravcova^1^†, Kirstyn Brunker^1,2^†, Paul Everest^1^, Manuel Fuentes^1^, Catherine Wilson^3^, Michael E. Murphy^3,4^, Taya Forde^1^

**Affiliations:**

^1^School of Biodiversity, One Health & Veterinary Medicine, University of Glasgow, Glasgow, United Kingdom

^2^MRC-University of Glasgow Centre for Virus Research, Glasgow, United Kingdom

^3^Department of Microbiology, NHS Greater Glasgow and Clyde, Glasgow Royal Infirmary, New Lister Building, Alexandra Parade, Glasgow, United Kingdom

^4^School of Medicine, Dentistry & Nursing, College of Medical, Veterinary & Life Sciences, Wolfson Medical School Building, University of Glasgow, Glasgow, United Kingdom

**Contribution:**

*MSIS corresponding author

†KO and †KB contributed equally

**Keywords:**

rapid diagnosis, selective host depletion, chemical host depletion, metagenomic next generation sequencing, Oxford Nanopore technologies, selective sequencing, adaptive sampling, bloodstream infection, antimicrobial resistance

**Running title:**

Rapid Nanopore sequencing for BSI diagnosis

**Supplementary Figures, tables and protocols**

**Tab. S1:** Double stranded DNA concentration of three BACT/ALERT negative (BCN01 to BCN03) and 30 BACT/ALERT positive blood culture (BCP) samples quantified by Qubit following M-15 CHDD and multiple displacement amplification with REPLI-g kit. As controls, six samples were also extracted directly and amplified similarly for 60 minutes without CHDD. Viability of bacterial following M-15 CHDD was confirmed by plating the supernatant. GC contents were calculated from assembled FASTA files using SeqKit (v.2.7.0).

| Sample | Accession | Organism | Culture | GC content (%) | Viability after M-15 CHDD | CHDD samples DNA (ng/µL) | No-CHDD samples DNA (ng/µL) |
| --- | --- | --- | --- | --- | --- | --- | --- |
| BCN01 | ERR13759391 | Not detected | Negative | N/A | N/A | 1.6 | 733 |
| BCN02 | ERR13759392 | Not detected | Negative | N/A | N/A | 2.8 | 767 |
| BCN03 | ERR13759393 | Not detected | Negative | N/A | N/A | 5.5 | 760 |
| BCP01 | ERR13759394 | *S. pyogenes* | Positive | 38.5 | Viable | 209 | 490 |
| BCP02 | ERR13759395 | *S. aureus* | Positive | 32.8 | Viable | 201 | 920 |
| BCP03 | ERR13759396 | *E. coli* | Positive | 50.8 | Viable | 350 | 940 |
| BCP04 | ERR13759397 | *S. pyogenes* | Positive | 38.5 | Viable | 291 | Not tested |
| BCP05 | ERR13759398 | *S. epidermidis* | Positive | 33.0 | Viable | 950 | Not tested |
| BCP06 | ERR13759399 | *S. epidermidis* | Positive | 33.0 | Viable | 620 | Not tested |
| BCP07 | ERR13759400 | *B. luteolum* | Positive | 67.8 | Viable | 17.4 | Not tested |
| BCP08 | ERR13759401 | *M. luteus* | Positive | 74.0 | Not viable | 9.44 | Not tested |
| BCP09 | ERR13759402 | *P. mirabilis* | Positive | 38.8 | Viable | 235 | Not tested |
| BCP10 | ERR13759403 | *S. agalactiae* | Positive | 35.5 | Viable | 77.8 | Not tested |
| BCP11 | ERR13759404 | *E. coli* | Positive | 50.8 | Viable | 1480 | Not tested |
| BCP12 | ERR13759405 | *E. coli* | Positive | 50.8 | Viable | 342 | Not tested |
| BCP13 | ERR13759406 | *K. michiganensis* | Positive | 55.5 | Viable | 1390 | Not tested |
| BCP14 | ERR13759407 | *E. coli* | Positive | 50.8 | Viable | 843 | Not tested |
| BCP15 | ERR13759408 | *K. variicola* | Positive | 57.3 | Viable | 1340 | Not tested |
| BCP16 | ERR13759409 | *S. agalactiae* | Positive | 35.5 | Viable | 325 | Not tested |
| BCP17 | ERR13759410 | *L. adecarboxylata* | Positive | 52.55 | Viable | 111 | Not tested |
| BCP18 | ERR13759411 | *E. coli* | Positive | 50.8 | Viable | 1170 | Not tested |
| BCP19 | ERR13759412 | *S. aureus* | Positive | 32.8 | Viable | 326 | Not tested |
| BCP20 | ERR13759413 | *S. maltophilia* | Positive | 66.7 | Viable | 18.1 | Not tested |
| BCP21 | ERR13759414 | *S. epidermidis* | Positive | 33.0 | Viable | 440 | Not tested |
| BCP22 | ERR13759415 | *E. faecalis* | Positive | 37.5 | Viable | 432 | Not tested |
| BCP23 | ERR13759416 | *K. pneumoniae* | Positive | 57.3 | Viable | 680 | Not tested |
| BCP24 | ERR13759417 | *P. mirabilis* | Positive | 38.7 | Viable | 860 | Not tested |
| BCP25 | ERR13759418 | *E. coli* | Positive | 50.8 | Viable | 1033 | Not tested |
| BCP26 | ERR13759419 | *S. gallolyticus* | Positive | 37.69 | Viable | 297 | Not tested |
| BCP27 | ERR13759420 | *S. aureus* | Positive | 32.8 | Viable | 228 | Not tested |
| BCP28 | ERR13759421 | *A. xylosoxidans* | Positive | 64.0 | Viable | 84 | Not tested |
| BCP29 | ERR13759422 | *S. epidermidis* | Positive | 33.0 | Viable | 406 | Not tested |
| BCP30 | ERR13759423 | *C. jejuni* | Positive | 30.3 | Viable | 348 | Not tested |

**Tab. S2:** Double stranded DNA concentration of three 8-hour culture enriched sterile blood (HB1 to HB3) versus ten spiked blood (1-10 CFU) samples following multiple displacement amplification with REPLI-g kit with and without M-15 chemical host DNA depletion (CHDD) measured by Qubit. Viability of bacteria following M-15 CHDD was confirmed by plating the supernatant on nutrient and blood agars. GC contents were calculated from assembled FASTA files using SeqKit (v.2.7.0).

| Sample | Organism | Culture | GC content (%) | Viability after M-15 CHDD | CHDD samples DNA (ng/µL) | No-CHDD samples DNA (ng/µL) |
| --- | --- | --- | --- | --- | --- | --- |
| HB1 | Not detected | Negative | NA | N/A | 4.2 | 652 |
| HB2 | Not detected | Negative | NA | N/A | 8.1 | 487 |
| HB3 | Not detected | Negative | NA | N/A | 4.18 | 513 |
| RESB1 | *S. aureus* | Positive | 32.8 | Viable | 131 | 510 |
| RESB2 | *E. faecium* | Positive | 37.8 | Viable | 122 | 611 |
| RESB3 | *S. pyogenes* | Positive | 38.5 | Viable | 140 | 377 |
| RESB4 | *S. agalactiae* | Positive | 35.6 | Viable | 177 | 647 |
| RESB5 | *S. pneumoniae* | Positive | 39.7 | Not viable | 83 | 550 |
| RESB6 | *P. aeruginosa* | Positive | 66.6 | Viable | 43 | 449 |
| RESB7 | *K. quasipneumoniae* | Positive | 57.2 | Viable | 213 | 714 |
| RESB8 | *A. baumannii* | Positive | 39 | Viable | 156 | 699 |
| RESB9 | *E. coli* | Positive | 50.8 | Viable | 248 | 892 |
| RESB10 | *P. mirabilis* | Positive | 38.88 | Viable | 233 | 621 |

**Tab. S3:** AMR gene profile (genes expected based on whole genome sequencing data) of the 8-hour culture-enriched blood samples chosen for sequencing (n=10). Following M-15 mNGS, sequencing reads were analysed with ResFinder to match and confirm the presence/absence of the same AMR determinants expected from the species initially utilised for spiking. Prediction accuracy of AMR (Match%) was determined comparing the ResFinder predicted phenotypic AST results of M-15 mNGS versus previously sequenced clinical/ATCC strains.

| **Sample** | **Accession** | **Organism** | **Source** | **Genes expected** | **Genes found (M-15)** | **Gene Match** | **Phenotype Match (%)** |
| --- | --- | --- | --- | --- | --- | --- | --- |
| RESB01 | ERR13759428 | *S. aureus* | NCTC 12493 | *mecA, ant(9)-Ia, erm(A), tet(K)* | *mecA, ant(9)-Ia, erm(A), tet(K)* | 4/4 | 100 |
| RESB02 | ERR13759429 | *E. faecium* | Clinical isolate | *aac(6')-II, ant(6)-Ia, aph(3')-III, aph(2'')-Id, erm(B), msr(C), lsa(E), tet(M)* | *aac(6')-II, ant(6)-Ia, aph(3')-III, aph(2'')-Id, erm(B), msr(C), lsa(E), tet(M)* | 8/8 | 100 |
| RESB03 | ERR13759430 | *S. pyogenes* | Clinical isolate | *-* | *-* | 0/0 | 100 |
| RESB04 | ERR13759431 | *S. agalactiae* | Clinical isolate | *mre(A)* | *mre(A)* | 1/1 | 100 |
| RESB05 | ERR13759432 | *S. pneumoniae* | ATCC 6303 | *-* | *-* | 0/0 | 100 |
| RESB06 | ERR13759433 | *P. aeruginosa* | ATCC 27853 | *aph(3')-IIb, blaPAO, blaOXA-396, fosA, catB7, crpP* | *catB7, crpP* | 2/6 | 86.9 |
| RESB07 | ERR13759434 | *K. quasipneumoniae* | ATCC 700603 | *ant(2'')-Ia, blaOKP-B, blaSHV-18, blaOXA-2, fosA, OqxA, OqxB, sul1* | *ant(2'')-Ia, blaOKP-B, blaSHV-18, blaOXA-2, fosA, OqxA, OqxB, sul1* | 8/8 | 100 |
| RESB08 | ERR13759435 | *A. baumannii* | Clinical isolate | *blaOXA-343, blaADC-25* | *blaOXA-343, blaADC-25* | 2/2 | 100 |
| RESB09 | ERR13759436 | *E. coli* | Clinical isolate | *aph(3'')-Ib, aph(6)-Id, blaTEM-1C, sul2* | *aph(3'')-Ib, aph(6)-Id, blaTEM-1C, sul2* | 4/4 | 100 |
| RESB10 | ERR13759437 | *P. mirabilis* | Clinical isolate | *tet(J)* | *tet(J)* | 1/1 | 100 |

**Tab. S4:** Primer, probe sequences and their reaction concentrations used in this study for detecting universal 16S (bacteria) and 18S Ribosomal ribonucleic acid-rRNA (host) genes and species-specific genes to identify three bacterial species, (*Escherichia coli* (EC), *Pseudomonas aeruginosa* (PA), and *Staphylococcus aureus* (SA)) used for initial spiking and benchmarking experiments.

| Genes | Primer/Probe name | Primer/Probe Conc (nM) | Sequence (5'-3') | Reference |
| --- | --- | --- | --- | --- |
|  |  |  |  |  |
| 16S rRNA | 16S rRNA_341_F | 400 | CCTACGGGAGGCAGCAG | Heravi et al ^4^. |
|  | 16S rRNA_534_R |  | ATTACCGCGGCTGCTGG |  |
|  | 16S rRNA_P | 100 | **FAM-**CCGCGTGWRTGAWGAAGGYCTTCG-**BHQ1** | This study |
| 18S rRNA | 18S rRNA_756_F | 400 | GGTGGTGCCCTTCCGTCA | Heravi et al ^4^. |
|  | 18S rRNA_877_R |  | CGATGCGGCGGCGTTATT |  |
|  | 18S rRNA_P | 100 | **Hex**- ACCCAAAGACTTTGGTTTCCCGGA- **BHQ1** | This study |
| EC_ybbW | EC_ybbW_F | 80 | TGATTGGCAAAATCTGGCCG | McQuillan et al ^5^. |
|  | EC_ybbW_R |  | GAAATCGCCCAAATCGCCAT |  |
|  | EC_ybbW_P | 40 | **FAM**-CCGCCGAAAACGATATAGATGCACGG- **BHQ1** |  |
| PA_GB722 | PA_GB722_F | 500 | GGCGTGGGTGTGGAAGTC | Lee et al ^6^. |
|  | PA_GB722_R |  | TGGTGAAGCAGAGCAGGTTCT |  |
|  | PA_GB722_P | 250 | **FAM**-TGCAGTGGAACGACA- **BHQ1** |  |
| SA_SaQuant | SA_SaQuant_F | 1000 | AACTACTAGGGGAGCCTAATRAT | Wood et al ^7^. |
|  | SA_SaQuant_R |  | GGTACTAACCAAATCAGGTCATAA |  |
|  | SA_SaQuant_P | 200 | **FAM**-TGGCTGAGATGAAYTGTTCAGACCC- **BHQ1** |  |

**Tab. S5:** Accuracy of species prediction with varying sequencing yield (100 Mbp to 10 Mbp) identified by subsampling the reads from 17 bacterial species from the 30 BACT/ALERT positive blood culture samples. Analysis was performed similarly using CZ ID with the same abundance threshold (20%) for all the species tested. Bacterial species highlighted red were missed with M-15 mNGS.

| Sample | Organism culture | M-15  (>100 Mbp) | M-15  (100 Mbp) | M-15  (50 Mbp) | M-15  (40 Mbp) | M-15  (30 Mbp) | M-15  (20 Mbp) | M-15  (10 Mbp) |
| --- | --- | --- | --- | --- | --- | --- | --- | --- |
| BCP01 | *Streptococcus pyogenes* | 1/1 | 1/1 | 1/1 | 1/1 | 1/1 | 1/1 | 1/1 |
| BCP02 | *Staphylococcus aureus* | 1/1 | 1/1 | 1/1 | 1/1 | 1/1 | 1/1 | 1/1 |
| BCP03 | *Escherichia coli* | 1/1 | 1/1 | 1/1 | 1/1 | 1/1 | 1/1 | 1/1 |
| BCP04 | *Streptococcus pyogenes* | 1/1 | 1/1 | 1/1 | 1/1 | 1/1 | 1/1 | 1/1 |
| BCP05 | ***Klebsiella oxytoca*** *and  Staphylococcus epidermidis* | 1/2 | 1/2 | 1/2 | 1/2 | 1/2 | 1/2 | 1/2 |
| BCP06 | *Staphylococcus epidermidis* | 1/1 | 1/1 | 1/1 | 1/1 | 1/1 | 1/1 | 1/1 |
| BCP07 | *Brevibacterium luteocum* | 1/1 | 1/1 | 1/1 | 1/1 | 1/1 | 1/1 | 1/1 |
| BCP08 | *Micrococcus luteus* | 1/1 | 1/1 | 1/1 | 1/1 | 1/1 | 1/1 | 1/1 |
| BCP09 | *Proteus mirabilis* | 1/1 | 1/1 | 1/1 | 1/1 | 1/1 | 1/1 | 1/1 |
| BCP10 | *Streptococcus agalactiae* | 1/1 | 1/1 | 1/1 | 1/1 | 1/1 | 1/1 | 1/1 |
| BCP11 | *Escherichia coli* | 1/1 | 1/1 | 1/1 | 1/1 | 1/1 | 1/1 | 1/1 |
| BCP12 | *Escherichia coli* | 1/1 | 1/1 | 1/1 | 1/1 | 1/1 | 1/1 | 1/1 |
| BCP13 | *Klebsiella oxytoca* | 1/1 | 1/1 | 1/1 | 1/1 | 1/1 | 1/1 | 1/1 |
| BCP14 | *Escherichia coli* | 1/1 | 1/1 | 1/1 | 1/1 | 1/1 | 1/1 | 1/1 |
| BCP15 | *Klebsiella pneumoniae* | 1/1 | 1/1 | 1/1 | 1/1 | 1/1 | 1/1 | 1/1 |
| BCP16 | *Streptococcus agalactiae* | 1/1 | 1/1 | 1/1 | 1/1 | 1/1 | 1/1 | 1/1 |
| BCP17 | *Leclercia adecarboxylata* | 1/1 | 1/1 | 1/1 | 1/1 | 1/1 | 1/1 | 1/1 |
| BCP18 | *Escherichia coli* | 1/1 | 1/1 | 1/1 | 1/1 | 1/1 | 1/1 | 1/1 |
| BCP19 | *Staphylococcus aureus* | 1/1 | 1/1 | 1/1 | 1/1 | 1/1 | 1/1 | 1/1 |
| BCP20 | *Stenotrophomonas maltophilia* | 1/1 | 1/1 | 1/1 | 1/1 | 1/1 | 1/1 | 1/1 |
| BCP21 | *Staphylococcus epidermidis* | 1/1 | 1/1 | 1/1 | 1/1 | 1/1 | 1/1 | 1/1 |
| BCP22 | *Enterococcus faecalis* | 1/1 | 1/1 | 1/1 | 1/1 | 1/1 | 1/1 | 1/1 |
| BCP23 | *Klebsiella pneumoniae* | 1/1 | 1/1 | 1/1 | 1/1 | 1/1 | 1/1 | 1/1 |
| BCP24 | *Proteus mirabilis and* ***Enterococcus faecalis*** | 1/2 | 1/2 | 1/2 | 1/2 | 1/2 | 1/2 | 1/2 |
| BCP25 | *Escherichia coli* | 1/1 | 1/1 | 1/1 | 1/1 | 1/1 | 1/1 | 1/1 |
| BCP26 | *Streptococcus gallolyticus* | 1/1 | 1/1 | 1/1 | 1/1 | 1/1 | 1/1 | 1/1 |
| BCP27 | *Staphylococcus aureus* | 1/1 | 1/1 | 1/1 | 1/1 | 1/1 | 1/1 | 1/1 |
| BCP28 | *Achromobacter denitrificans* | 1/1 | 1/1 | 1/1 | 1/1 | 1/1 | 1/1 | 1/1 |
| BCP29 | *Staphylococcus epidermidis* | 1/1 | 1/1 | 1/1 | 1/1 | 1/1 | 1/1 | 1/1 |
| BCP30 | *Campylobacter jejuni* | 1/1 | 1/1 | 1/1 | 1/1 | 1/1 | 1/1 | 1/1 |
| N = 30 | *N = 32* | 30/32 | 30/32 | 30/32 | 30/32 | 30/32 | 30/32 | 30/32 |

**Tab. S6:** Accuracy of species prediction with varying sequencing yield (100 Mbp to 10 Mbp) identified by subsampling the reads from 10 rapid (8-hours) culture enriched spiked blood samples. Analysis was performed similarly using CZ ID with the same abundance threshold (20%) for all the species tested.

| Sample | Organism culture | M-15  (>100 Mbp) | M-15  (100 Mbp) | M-15  (50 Mbp) | M-15  (40 Mbp) | M-15  (30 Mbp) | M-15  (20 Mbp) | M-15  (10 Mbp) |
| --- | --- | --- | --- | --- | --- | --- | --- | --- |
| RESB01 | *Staphylococcus aureus* | 1/1 | 1/1 | 1/1 | 1/1 | 1/1 | 1/1 | 1/1 |
| RESB02 | *Enterococcus faecium* | 1/1 | 1/1 | 1/1 | 1/1 | 1/1 | 1/1 | 1/1 |
| RESB03 | *Streptococcus pyogenes* | 1/1 | 1/1 | 1/1 | 1/1 | 1/1 | 1/1 | 1/1 |
| RESB04 | *Streptococcus agalactiae* | 1/1 | 1/1 | 1/1 | 1/1 | 1/1 | 1/1 | 1/1 |
| RESB05 | *Staphylococcus pneumoniae* | 1/1 | 1/1 | 1/1 | 1/1 | 1/1 | 1/1 | 1/1 |
| RESB06 | *Pseudomonas aeruginosa* | 1/1 | 1/1 | 1/1 | 1/1 | 1/1 | 1/1 | 1/1 |
| RESB07 | *Klebsiella quasipneumoniae* | 1/1 | 1/1 | 1/1 | 1/1 | 1/1 | 1/1 | 1/1 |
| RESB08 | *Acinetobacter baumannii* | 1/1 | 1/1 | 1/1 | 1/1 | 1/1 | 1/1 | 1/1 |
| RESB09 | *Escherichia coli* | 1/1 | 1/1 | 1/1 | 1/1 | 1/1 | 1/1 | 1/1 |
| RESB10 | *Proteus mirabilis* | 1/1 | 1/1 | 1/1 | 1/1 | 1/1 | 1/1 | 1/1 |
| N = 10 | *N = 10* | 10/10 | 10/10 | 10/10 | 10/10 | 10/10 | 10/10 | 10/10 |

**Tab. S7:** AMR gene profiles profiles predicted with varying sequencing yield (>100 Mbp to 10 Mbp/sample) using subsampled reads from the 30 BACT/ALERT positive blood culture samples. Gene counts are shown for each yield to show how detection varies across decreasing sequencing outputs.

| Sample | Organism culture | M-15  (>100 Mbp) | M-15  (100 Mbp) | M-15  (50 Mbp) | M-15  (40 Mbp) | M-15  (30 Mbp) | M-15  (20 Mbp) | M-15  (10 Mbp) |
| --- | --- | --- | --- | --- | --- | --- | --- | --- |
| BCP01 | *Streptococcus pyogenes* | 0 | 0/0 | 0/0 | 0/0 | 0/0 | 0/0 | 0/0 |
| BCP02 | *Staphylococcus aureus* | 2 | 2/2 | 2/2 | 2/2 | 2/2 | 1/2 | 0/2 |
| BCP03 | *Escherichia coli* | 0 | 0/0 | 0/0 | 0/0 | 0/0 | 0/0 | 0/0 |
| BCP04 | *Streptococcus pyogenes* | 0 | 0/0 | 0/0 | 0/0 | 0/0 | 0/0 | 0/0 |
| BCP05 | ***Klebsiella oxytoca*** *and  Staphylococcus epidermidis* | 7 | 7/7 | 7/7 | 7/7 | 6/7 | 4/7 | 4/7 |
| BCP06 | *Staphylococcus epidermidis* | 3 | 3/3 | 3/3 | 3/3 | 3/3 | 3/3 | 1/3 |
| BCP07 | *Brevibacterium luteocum* | 0 | 0/0 | 0/0 | 0/0 | 0/0 | 0/0 | 0/0 |
| BCP08 | *Micrococcus luteus* | 0 | 0/0 | 0/0 | 0/0 | 0/0 | 0/0 | 0/0 |
| BCP09 | *Proteus mirabilis* | 9 | 9/9 | 8/9 | 6/9 | 2/9 | 4/9 | 2/9 |
| BCP10 | *Streptococcus agalactiae* | 1 | 1/1 | 1/1 | 1/1 | 1/1 | 1/1 | 0/1 |
| BCP11 | *Escherichia coli* | 0 | 0/0 | 0/0 | 0/0 | 0/0 | 0/0 | 0/0 |
| BCP12 | *Escherichia coli* | 13 | 13/13 | 7/13 | 4/13 | 3/13 | 1/13 | 0/13 |
| BCP13 | *Klebsiella oxytoca* | 2 | 2/2 | 2/2 | 2/2 | 2/2 | 0/2 | 0/2 |
| BCP14 | *Escherichia coli* | 0 | 0/0 | 0/0 | 0/0 | 0/0 | 0/0 | 0/0 |
| BCP15 | *Klebsiella pneumoniae* | 5 | 5/5 | 6/5 | 5/5 | 1/5 | 2/5 | 0/5 |
| BCP16 | *Streptococcus agalactiae* | 2 | 3/2 | 2/2 | 2/2 | 2/2 | 0/2 | 1/2 |
| BCP17 | *Leclercia adecarboxylata* | 0 | 0/0 | 0/0 | 1/0 | 0/0 | 0/0 | 0/0 |
| BCP18 | *Escherichia coli* | 0 | 0/0 | 0/0 | 0/0 | 0/0 | 0/0 | 0/0 |
| BCP19 | *Staphylococcus aureus* | 11 | 1/11 | 0/11 | 0/11 | 1/11 | 1/11 | 0/11 |
| BCP20 | *Stenotrophomonas maltophilia* | 1 | 1/1 | 1/1 | 0/1 | 0/1 | 0/1 | 0/1 |
| BCP21 | *Staphylococcus epidermidis* | 9 | 9/9 | 5/9 | 7/9 | 7/9 | 8/9 | 6/9 |
| BCP22 | *Enterococcus faecalis* | 3 | 2/3 | 2/3 | 3/3 | 3/3 | 2/3 | 1/3 |
| BCP23 | *Klebsiella pneumoniae* | 13 | 13/13 | 13/13 | 6/13 | 6/13 | 5/13 | 3/13 |
| BCP24 | *Proteus mirabilis and* ***Enterococcus faecalis*** | 1 | 1/1 | 1/1 | 1/1 | 1/1 | 0/1 | 0/1 |
| BCP25 | *Escherichia coli* | 4 | 4/4 | 4/4 | 4/4 | 4/4 | 4/4 | 1/4 |
| BCP26 | *Streptococcus gallolyticus* | 3 | 2/3 | 2/3 | 3/3 | 3/3 | 2/3 | 2/3 |
| BCP27 | *Staphylococcus aureus* | 9 | 6/9 | 4/9 | 3/9 | 2/9 | 1/9 | 1/9 |
| BCP28 | *Achromobacter denitrificans* | 1 | 0/1 | 1/1 | 0/1 | 0/1 | 0/1 | 0/1 |
| BCP29 | *Staphylococcus epidermidis* | 11 | 11/11 | 11/11 | 11/11 | 11/11 | 8/11 | 5/11 |
| BCP30 | *Campylobacter jejuni* | 2 | 1/2 | 1/2 | 1/2 | 1/2 | 1/2 | 1/2 |
| N = 30 | N = 32 | 112 | 96/112 | 83/112 | 72/112 | 61/112 | 48/112 | 28/112 |

**Note*** Phenotypic predictions from M-15 (>100 Mbp) column showed 90.27% categorical agreement, with 9.28% Major Error and 18.42% Very Major Error compared to culture based AST results (Figure 4D)

**Tab. S8:** AMR gene profiles predicted with varying sequencing yield (>50 Mbp to 10 Mbp/sample) using the subsampled reads from 10 rapid culture enriched (8-hours) spiked blood samples. Gene counts are shown for each yield to show how detection varies across decreasing sequencing outputs.

| Sample | Organism culture | Genes expected | >50 Mbp | 40 Mbp | 30 Mbp | 20 Mbp | 10 Mbp |
| --- | --- | --- | --- | --- | --- | --- | --- |
| RESB01 | *Staphylococcus aureus* | 4 | 4/4 | 4/4 | 4/4 | 2/4 | 2/4 |
| RESB02 | *Enterococcus faecium* | 8 | 8/8 | 6/8 | 6/8 | 6/8 | 5/8 |
| RESB03 | *Streptococcus pyogenes* | 0 | 0/0 | 0/0 | 0/0 | 0/0 | 0/0 |
| RESB04 | *Streptococcus agalactiae* | 1 | 1/1 | 1/1 | 1/1 | 0/1 | 0/1 |
| RESB05 | *Staphylococcus pneumoniae* | 0 | 0/0 | 0/0 | 0/0 | 0/0 | 0/0 |
| RESB06 | *Pseudomonas aeruginosa* | 6 | 2/6 | 2/6 | 2/6 | 2/6 | 0/6 |
| RESB07 | *Klebsiella quasipneumoniae* | 8 | 8/8 | 8/8 | 5/8 | 3/8 | 1/8 |
| RESB08 | *Acinetobacter baumannii* | 2 | 2/2 | 2/2 | 1/2 | 1/2 | 0/2 |
| RESB09 | *Escherichia coli* | 4 | 4/4 | 4/4 | 0/4 | 1/4 | 0/4 |
| RESB10 | *Proteus mirabilis* | 1 | 1/1 | 1/1 | 1/1 | 1/1 | 0/1 |
| Total | *N = 10* | 34 | 30/34 | 28/34 | 20/34 | 16/34 | 8/34 |

**Supplement Protocol: M-15 mNGS**

Consumables

| **Name** | **Concentration/units** | **Commercial source** | **Comments** |
| --- | --- | --- | --- |
| Saponin | Final concentration 2.25% | Tokyo chemical industry, Cat: S0019 | To make a 4.5% or 2.25% w/V solution, dissolve 4.5/2.25 grams of Saponin into 50 mL sterile nuclease free H_2_O. Mix thoroughly until dissolved. Adjust the volume to 100 mL Sterilize using 0.2 μm filter, and store at 4 °C for up to 3 weeks. |
| NaCl | 5M | Promega, Cat: V4221 | NA |
| DNase I | 300 units/μL | Invitrogen, Cat: 18047019 | NA |
| DNase I buffer | 10x solution | NEB, Cat: B0303SVIAL | 10x buffer = 10 mM Tris HCl, 2.5 mM MgCl_2_, 0.5 mM CaCl_2_ |
| Trypsin-EDTA | 0.05%, phenol red | ThermoFisher Cat: 25300054 | NA |
| Molecular grade H20 | NA | Qiagen, Cat: 129114 | NA |
| DPBS | 1x solution | ThermoFisher Cat: 14190144 | Without calcium, magnesium, or phenol |
| T7 Endonuclease I | NA | NEB, Cat: M0302S or M0302L | NA |
| DNA binding beads | NA | Aline biosciences, Cat: C-1003-50 | NA |
| Molecular grade ethanol | NA | Sigmaaldrich, Cat: 51976-500ML-F | NA |
| REPLI-g Single Cell Kit | NA | Qiagen, Cat: 150343 or 150345 | NA |
| Qubit dsDNA Be assay Kit | NA | ThermoFisher, Cat: Q32853 | NA |
| Qubit assay tubes | NA | ThermoFisher, Cat: Q32856 | NA |
| Rapid Barcoding Kit | NA | ONT, Cat: SQK-RBK004 | Required for the rapid sequencing protocol |
| Long Fragment Buffer Expansion | NA | ONT, Cat: EXP-LFB001 | Required for the rapid sequencing protocol |
| Ligation Sequencing Kit | NA | ONT, Cat: SQK-LSK109 | Required for the high yield protocol |
| Native Barcoding Expansion Kit | NA | ONT, Cat: EXP-NBD196 | Required for the high yield protocol |
| NEBNext Ultra II End Repair/dA-Tailing Module | NA | NEB, Cat: E7546L | Required for the high yield protocol |
| Blunt/TA Ligase Master Mix | NA | NEB, Cat: M0367L | Required for the high yield protocol |
| NEBNext Quick Ligation Module | NA | NEB, Cat: E6056L | Required for the high yield protocol |
| Flow Cell Priming Kit | NA | ONT, Cat: EXP-FLP002 | Required for both protocols |
| Flow Cell Wash Kit | NA | ONT, Cat: EXP-WSH004 | Required for both protocols |
| ONT Flow Cell R9.4.1 | NA | ONT, Cat: FLO-MIN106 or FLO-FLG001 | Required for both protocols |
| Magnetic rack/plate holder | NA | ThermoFisher, Cat: 12331D and 12321D | NA |
| PCR Tube Strip with Attached Flat Caps | NA | ThermoFisher, Cat: AB2000 | NA |
| 1.5 ml DNA LoBind tubes | NA | Eppendorf, Cat: 0030108051-250EA | Use LoBind tubes |
| 2 ml round bottom tubes | NA | Starlab, Cat: S1620-2700-C or Eppendorf, Cat: 0030120094 | Avoid using LoBind tubes |
| Pipettes and pipette tips P2, P10, P20, P100, P200, P1000, Multichannel | NA | Any | NA |
| Centrifuge /Microplate centrifuge | NA | Any | NA |
| Vortex mixer | NA | Any | NA |
| Thermal cycler | NA | Any | NA |
| Heating block for 1.5/2 mL tubes | NA | Any | NA |
| Sequencing device | NA | Flongle, MinION Mk1B,  GridION | NA |
| Device for live basecalling | NA | GridION or  A powerful laptop | A laptop with minimum 8 Gb GPU (RTX 3060 or higher), 16 GB Ram, 1 Tb M.2 NVMe SSD |

**Disclaimer:** Some parts of the following protocol have been copied directly from the manufacturer’s official website or from the kit manual. Therefore, all the proprietary information mentioned below remains the sole property of the kit’s manufacturer.

Before sample preparation

It is important to divide all reagents into smaller aliquots to avoid multiple freeze-thaw cycles and reduce the likelihood of cross-contamination.

Warm up 0.05% trypsin EDTA at 37°C. Prepare 1x DNase I buffer and store in the fridge before use. Make a fresh batch of REPLI-g Master Mix following manufacturer’s instructions. Take out aliquots of Buffer D2, Stop Solution, and any other reagents from the freezer, and store in the refrigerator or on ice until needed. Thaw all the reagents at room temperature or on ice as per manufacturers recommendation.

This protocol can be used on BD BACTEC (Becton, Dickinson and Company, NJ, USA) or any blood culture (FPBC) samples flagged positive with an automated culture system. Protocol M-15 mNGS can also be applied to rapid culture enriched blood samples (REBC; for example, 1-10 mL patient blood samples incubated for 8 hours in BD BACTEC media bottles), once the enriched sampled have at least 10^3^ CFU/mL bacterial concentration. FPBC samples can be used directly for CHDD; however, REBC samples need to be pre-processed to concentrate the sample before continuing with this protocol. It is crucial to use fresh samples for CHDD, as frozen or improperly stored samples can lead to the lysis of bacterial cells. This may lead to a considerable loss of bacterial DNA during CHDD.

Pre-processing REBC samples

Transfer 1.5 mL REBC samples aseptically to a 2 mL round bottom microcentrifuge tube and spin at 8,000x*g* for 5 minutes. Slowly aspirate the supernatant without disturbing the pellet and gently resuspend the pellet in 800 μL 2.25% saponin. Proceed to the step 2 of CHDD protocol below.

Note* It is important to use round/wide bottom 2 mL microcentrifuge tubes to minimize bacterial cell loss while aspirating the supernatant during the initial washing steps.

Host depletion

1. Add 400 μL FPBC sample to 400 μL 4.5% saponin (final saponin concentration will be 2.25% after mixing) in a 2 mL wide bottom sterile microcentrifuge tube and gently pipette mix for at least 4 to 5 times.

2. Add 1 μL DNase I (300 units/μL) to the REBC/FPBC sample, pulse vortex at low speed for 5 seconds and incubate the sample at 25°C in a heating block with a built-in shaker for 10 minutes at 800 rpm.

Note* Adding DNase I decreases the viscosity of the sample by degrading a substantial amount of DNA (desirably from the host) after selective host cell lysis. The DNA released from the host cells can be extremely sticky, and often adhere to pipette tips or microfuge tubes. As a result, there's a risk of losing desired/targeted bacterial cells because they might adhere to the sticky host DNA and get affixed to the pipette tips and/or microcentrifuge tube.

3. Perform a quick spin and add 800 μL molecular grade water to the sample. Pipette mix 4 to 5 times and incubate for 1 minute at room temperature.

4. Add 44 μL 5 M molecular grade NaCl (final concentration is 137 mM; same as PBS) to the sample, invert the tubes 3 to 4 times to mix and centrifuge at 10,000x *g* for 5 minutes.

5. Carefully remove the supernatant without touching or disturbing the pellet and resuspend in 1 mL molecular grade 1x Dulbecco's Phosphate Buffered Saline (DPBS, Without calcium, magnesium, or phenol).

6. Centrifuge the sample at 10,000x *g* for 3 minutes and carefully remove the supernatant without touching or disturbing the pellet.

7. Resuspend the pellet in 398 μL 1x DNase I buffer, add 2 μL DNase I (300 units/μL) to the sample and incubate at 37°C with shaking at 800 RPM for 15 minutes.

8. Following incubation, add 1 mL 0.05% trypsin EDTA directly to the sample, pipette mix at least 8 to 10 times and incubate at room temperature for 1 minute.

Note* Some bacterial cells may form aggregates in the sample, so adding 0.05% trypsin EDTA can separate the cells and ensure an even distribution for subsequent procedures. The trypsinisation step does not affect bacterial viability and/or cell wall integrity.

9. Repeat step 6 and resuspend the pellet in 1 mL 1x DPBS.

Note* This is the end of host depletion with M-15. After this, 4 μL of host depleted bacterial cell material in DPBS from step 9 can be used directly for alkaline lysis and whole genome amplification (WGA; protocol 1) using the REPLI-g Single Cell Kit.

DNA extraction and Whole Genome Amplification

1. Prepare sufficient Buffer D2 in a PCR tube for the total number of 12 reactions.

| **Reagent** | **Volume (μL)** |
| --- | --- |
| DTT, 1M | 3 |
| Buffer DLB | 33 |
| Total | 36 |

Note* Buffer D2 should not be stored longer than 2 months at -20 °C.

1. Place 4 µL cell material into a PCR tube containing 3 µL buffer D2. Mix by flicking the tube and centrifuge briefly.
2. Incubate at 65°C for 10 min with heated lid on and then add 3 µL Stop Solution. Mix by flicking the tube and centrifuge briefly. Prepare a master mix according to the instruction below.

| **Reagent** | **Volume (μL)** |
| --- | --- |
| REPLI-g sc Reaction Buffer | 14 |
| REPLI-g sc DNA Polymerase | 1 |
| Total | 15 |

Note* Avoid using H_2_O for preparing the master mix.

1. For each reaction, add 5 µL DNA from step 12 and incubate at 30°C for 1 hour with heated lid off.
2. Inactivate REPLI-g sc DNA Polymerase by heating the sample for 3 min at 65°C with heated lid on.

Note* After this step amplified host depleted DNA can be diluted 5-fold in molecular grade H_2_O, measured with Qubit Br assay and used directly for debranching and subsequently sequencing library preparation.

Debranching and bead cleanup

1. In a clean 0.2 mL PCR tube, mix the reagents for debranching in the following order.

| **Reagent** | **Volume (μL)** |
| --- | --- |
| 600 ng of amplified DNA | To make 30 μL |
| NEBuffer 2 | 3 |
| T7 Endonuclease I | 1.5 |
| Nuclease free H_2_O | 25.5- To make 30 μL |
| Total | 30 |

1. Mix the reagents by flicking the tube and centrifuge briefly. Incubate the reaction for 10 minutes at 37°C.
2. Resuspend the DNA binding beads (e.g., AMPure XP or Aline biosciences) by vortexing.
3. Add 15 µL of the bead suspension (0.5x) to the DNA sample and mix by pipetting.
4. Incubate for 5 minutes at room temperature.
5. Spin down and pellet the sample on a magnetic rack/plate until supernatant is clear and colourless. Keep the tube on the magnet, and pipette off the supernatant.
6. Keeping the tube on the magnet, wash the beads with 200 µL of freshly prepared 70% ethanol without touching/disturbing the pellet. Remove the ethanol carefully using a pipette and discard.
7. Repeat the previous ethanol washing step.
8. Spin down and put the tube back on the magnetic rack/plate. Pipette off any residual ethanol. Open the lid and allow the tubes to dry for ~30 seconds, but do not dry the pellet to the point of cracking.
9. Remove the tube from the magnetic rack/plate and resuspend the pellet in 15 µL nuclease-free water. Incubate for 2 min at room temperature.
10. Pellet the beads on a magnet until the eluate is clear and colourless.
11. Remove and retain 12 µL of eluate into a clean 0.2 mL PCR tube.

Note* This is the end of debranching and DNA cleanup. After this step, cleaned and debranched DNA products can be used for library preparation with Rapid Barcoding (rapid but low yield) or Ligation sequencing kit (highest yield).

Rapid barcoding (low-medium yield protocol)

Barcoding

1. Combine the following components per PCR tube:

| **Reagent** | **Volume (μL)** |
| --- | --- |
| Debranched, cleaned DNA (400 ng) | 7.5 |
| Fragmentation Mix (RB01-12) | 2.5 |
| Total | 10 |

1. Mix the reagents by flicking the tube and spin briefly.
2. Incubate the samples at 30°C in a thermal cycler for 1 minute and 80°C for 1 minutes with heated lid off.
3. Spin and pool all the rapid barcoded DNA in 1.5 mL Eppendorf DNA LoBind tube to make 1,200 ng in a volume of 50 µL. Example, for 6 samples, take 200 ng (200 x 6 = 1,200 ng) DNA per sample.
4. Resuspend the DNA binding beads (e.g., AMPure XP or Aline biosciences) by vortexing.
5. Add 25 µL (0.5x) of resuspended beads to the sample and mix by flicking the tube multiple times.
6. Incubate the tube for 5 minutes at room temperature.
7. Spin down the sample and pellet on a magnetic rack/plate until the supernatant is clear and colourless. Keep the tube on the magnet, and carefully pipette off the supernatant.
8. Wash the beads with 250 μl long Fragment Buffer (LFB) without touching/disturbing the pellet. Remove LFB carefully using a pipette and discard.

Note* Flick the beads to resuspend, spin and put back on magnet and remove supernatant using a pipette. Long Fragment Buffer Expansion (EXP-LFB001) can be purchased separately from ONT.

1. Repeat the previous LFB washing step.
2. Spin down and place the tube back on the magnet. Pipette off any residual supernatant. Allow to dry for ~30 seconds, but do not dry the pellet to the point of cracking.
3. Remove the tube from the magnetic rack/plate and resuspend the pellet in 22 µL Elution Buffer (EB). Spin down briefly and incubate for 2 minutes at room temperature.
4. Pellet the beads on a magnetic rack/plate until the eluate is clear and colourless, for at least 1 minute.
5. Remove and retain 20 µL of eluate containing the DNA library into a clean 1.5 mL Eppendorf DNA LoBind tube.

Rapid adapter ligation and loading

1. Quantify 1 µL rapid barcoded and cleaned DNA using a Qubit fluorometer.
2. Add 1 µL rapid adaptor (RAP) to 10 µL of barcoded DNA in EB in a PCR tube, flick multiple times to mix, spin briefly and incubate for 10 minutes at 20°C with the heated lid off.
3. Load 100 ng (51.36 fmol; considering 3kb product size) of final prepared library onto a flow cell following manufacturers recommendation.

**Ligation sequencing (high yield protocol)**

**End prep**

1. Combine the following components per PCR tube:

| **Reagent** | **Volume (μL)** |
| --- | --- |
| Ultra II End-prep reaction buffer | 0.875 |
| Ultra II End-prep enzyme mix | 0.75 |
| Debranched, cleaned DNA | 12 |
| Total | 13.625 |

1. Mix gently by flicking the tube, and spin down.
2. Using a thermal cycler, incubate at 20°C for 5 minutes with heated lid off.
3. Incubate at 65°C for 5 minutes with heated lid on.

**Native barcode ligation and cleanup**

1. Combine the following components per PCR tube:

| **Reagent** | **Volume (μL)** |
| --- | --- |
| Native Barcode | 1.25 |
| Blunt/TA Ligase Master Mix | 6 |
| End prepped DNA | 6 |
| Total | 13.25 |

1. Mix gently by flicking the tube, and spin down.
2. Incubate the reaction for 10 minutes at 20°C with heated lid off.
3. Resuspend the AMPure XP beads by vortexing.
4. Add 13.25 µL of resuspended AMPure XP beads to the reaction and mix by pipetting.
5. Incubate for 5 minutes at room temperature.
6. Spin down the sample and pellet on a magnet until supernatant is clear and colourless. Keep the tube on the magnet, and pipette off the supernatant.
7. Keep the tube on the magnet and wash the beads with 200 µL of freshly prepared 70% ethanol without disturbing the pellet. Remove the ethanol using a pipette and discard.
8. Repeat the previous step.
9. Spin down and place the tube back on the magnet. Pipette off any residual supernatant. Allow to dry for ~30 seconds, but do not dry the pellet to the point of cracking.
10. Remove the tube from the magnetic plate and resuspend pellet in 15 µL nuclease-free water. Incubate for 2 min at room temperature.
11. Pellet the beads on a magnet until the eluate is clear and colourless.
12. Remove and retain 12 µL of eluate into a clean PCR tube.

Measure DNA concentration with Qubit and pool all the barcoded DNA in 1.5 mL Eppendorf DNA LoBind tube to make 350 ng in a volume of 30 µL H2O. Example, for 6 samples, take 58.33 ng (58.33 x 6 = 350 ng) DNA per sample.

**Adapter ligation and cleanup and loading**

1. Combine the following components:

| **Reagent** | **Volume (μL)** |
| --- | --- |
| Pooled barcoded DNA (350 ng) | 30 |
| Adapter Mix II (AMII) | 5 |
| Ligation Buffer (LNB) | 10 |
| Quick T4 DNA Ligase | 5 |
| Total | 50 |

1. Ensure the components are thoroughly mixed by pipetting, and spin down.
2. Incubate the reaction for 15 minutes at 20°C.
3. Resuspend the AMPure XP beads by vortexing.
4. Add 25 µL (0.5x) of resuspended AMPure XP beads to the reaction and mix by pipetting.
5. Incubate for 5 minutes at room temperature.
6. Spin down the sample and pellet on a magnet until supernatant is clear and colourless. Keep the tube on the magnet, and pipette off the supernatant.
7. Wash the beads by adding 250 μl long Fragment Buffer (LFB).

Note* Do not flick the beads to resuspend. Just gently wash and remove the supernatant using a pipette and discard.

1. Repeat the previous step.
2. Spin down and place the tube back on the magnet. Pipette off any residual supernatant. Allow to dry for ~30 seconds, but do not dry the pellet to the point of cracking.
3. Remove the tube from the magnetic rack and resuspend the pellet in 17 µL Elution Buffer (EB). Spin down and incubate for 2 minutes at room temperature.
4. Pellet the beads on a magnet until the eluate is clear and colourless, for at least 1 minute.
5. Remove and retain 15 µL of eluate containing the DNA library into a clean 1.5 mL Eppendorf DNA LoBind tube.
6. Quantify 1 µL of adapter ligated and barcoded DNA using a Qubit fluorometer.
7. Load 100 ng (51.36 fmol; considering 3kb) of final prepared library onto a flow cell following manufacturers recommendation.

Note* The typical library size after this stage is around 3 kb. However, users can verify this by running the library on a gel or TapeStation for a few batches. Once the average expected library size is determined (sample or site-wise), there will be no need to repeat this for every batch of samples.

For loading sequencing library or washing flow cells, follow the manufacturers recommended protocols.

**References**

1. Charalampous, T. et al. Nanopore metagenomics enables rapid clinical diagnosis of bacterial lower respiratory infection. *Nat Biotechnol* **37**, 783-792 (2019).

2. Trung, N.T. et al. Enrichment of bacterial DNA for the diagnosis of blood stream infections. *BMC Infect Dis* **16**, 235 (2016).

3. Marotz, C.A. et al. Improving saliva shotgun metagenomics by chemical host DNA depletion. *Microbiome* **6**, 1-9 (2018).

4. Heravi, F.S., Zakrzewski, M., Vickery, K. & Hu, H. Host DNA depletion efficiency of microbiome DNA enrichment methods in infected tissue samples. *J Microbiol Methods* **170**, 105856 (2020).

5. McQuillan, J.S. & Wilson, M.W. 'Ready Mixed', improved nucleic acid amplification assays for the detection of Escherichia coli DNA and RNA. *J Microbiol Methods* **165**, 105721 (2019).

6. Lee, C.S., Wetzel, K., Buckley, T., Wozniak, D. & Lee, J. Rapid and sensitive detection of Pseudomonas aeruginosa in chlorinated water and aerosols targeting gyrB gene using real-time PCR. *J Appl Microbiol* **111**, 893-903 (2011).

7. Wood, C. et al. SaQuant: a real-time PCR assay for quantitative assessment of Staphylococcus aureus. *BMC Microbiol* **21**, 174 (2021).
