# Supplementary material for: Rapid and modular workflows for same-day sequencing-based detection of bloodstream infections and antimicrobial resistance determinants using culture-enriched samples
Source: Microbiol Spectr. 2026 Jun 15;14(7):e03240-25. doi: 10.1128/spectrum.03240-25 (PMC13339894; doi:10.1128/spectrum.03240-25)
Supplement: Reviewer comments [file reviewer-comments.pdf]

AMERICAN SOCIETY FOR MICROBIOLOGY | Microbiology Spectrum

Rapid and modular workflows for same-day sequencing-based detection of bloodstream infections and antimicrobial resistance determinants using culture-enriched samples

Mohammad Sajib, Katarina Oravcova, Kirstyn Brunker, Paul Everest, Manuel Fuentes, Catherine Wilson, Michael Murphy, and Taya Forde

Corresponding Author(s): Mohammad Sajib, University of Glasgow

Review Timeline:

Submission Date:	November 11, 2025
Editorial Decision:	December 24, 2025
Revision Received:	January 19, 2026
Accepted:	March 20, 2026

Editor: Sadjia Bekal

Reviewer(s): Disclosure of reviewer identity is with reference to reviewer comments included in decision letter(s). The following individuals involved in review of your submission have agreed to reveal their identity: Natalie Ring (Reviewer #1)

Transaction Report:

(Note: With the exception of the correction of typographical or spelling errors that could be a source of ambiguity, letters and reports are not edited. The original formatting of letters and referee reports may not be reflected in this compilation.)

DOI: https://doi.org/10.1128/spectrum.03240-25

Re: Spectrum03240-25 (Rapid and modular workflows for same-day sequencing-based detection of bloodstream infections and antimicrobial resistance determinants using culture-enriched samples)

Dear Dr. Mohammad Saiful Islam Sajib:

Thank you for the privilege of reviewing your work. Below you will find my comments, instructions from the Spectrum editorial office, and the reviewer comments.

Please return the manuscript within 60 days; if you cannot complete the modification within this time period, please contact me. If you do not wish to modify the manuscript and prefer to submit it to another journal, notify me immediately so that the manuscript may be formally withdrawn from consideration by Spectrum.

Revision Guidelines
To submit your modified manuscript, log into the submission site at https://spectrum.msubmit.net/cgi-bin/main.plex. Go to Author Tasks and click the appropriate manuscript title to begin. The information you entered when you first submitted the paper will be displayed; update this as necessary. Note the following requirements:

• Upload point-by-point responses to the issues raised by the reviewers in a file named "Response to Reviewers," NOT in your cover letter.
• Upload a compare copy of the manuscript (without figures) as a "Marked-Up Manuscript" file.
• Upload a clean .DOC/.DOCX version of the revised manuscript and remove the previous version.
• Each figure must be uploaded as a separate, editable, high-resolution file (TIFF or EPS preferred), and any multipanel figures must be assembled into one file.
• Any supplemental material intended for posting by ASM should be uploaded with their legends separate from the main manuscript. You can combine all supplemental material into one file (preferred) or split it into a maximum of 10 files with all associated legends included.

For complete guidelines on revision requirements, see our Submission and Review Process webpage. Submission of a paper that does not conform to guidelines may delay acceptance of your manuscript.

Data availability: ASM policy requires that data be available to the public upon online posting of the article, so please verify all links to sequence records, if present, and make sure that each number retrieves the full record of the data. If a new accession number is not linked or a link is broken, provide Spectrum production staff with the correct URL for the record. If the accession numbers for new data are not publicly accessible before the expected online posting of the article, publication may be delayed; please contact production staff (Spectrum@asmusa.org) immediately with the expected release date.

Publication Fees: For information on publication fees and which article types are subject to charges, visit our website. If your manuscript is accepted for publication and any fees apply, you will be contacted separately about payment during the production process; please follow the instructions in that e-mail. Arrangements for payment must be made before your article is published.

ASM Membership: Corresponding authors may join or renew ASM membership to obtain discounts on publication fees. Need to upgrade your membership level? Please contact Customer Service at Service@asmusa.org.

The ASM Journals program strives for constant improvement in our submission and publication process. Please tell us how we can improve your experience by taking this quick Author Survey.

Thank you for submitting your paper to Spectrum.

Sincerely,
Sadjia Bekal
Editor
Microbiology Spectrum

Reviewer #1 (Comments for the Author):

In the manuscript "Rapid and modular workflows for same-day sequencing-based detection of bloodstream infections and antimicrobial resistance determinants using culture-enriched samples", Sajib et al. present the development of a workflow to detect bacterial species and AMR determinants in metagenomic sequencing data from blood samples, including thorough validation of various steps such as host depletion, incubation-based enrichment and sequencing library preparation. This is an

impressive manuscript which provides detailed descriptions and justification of the methods used (including in the supplementary methods). Although long, I believe the level of detail provided in the manuscript is valuable for others working in the field of clinical metagenomics.

The aims and methods used in the study are valid, the introduction is well-written, and the discussion applies a suitable level of realistic criticism of the current applicability of the workflows for clinical diagnostics. Bearing in mind the previous revisions, I have no additional comments.

Reviewer #2 (Comments for the Author):

1.----How was fold depletion calculated? Although it is written in the figure legends, it should be mentioned in the methods section.

2.----The statistical significance between the host depletion is missing.

3.----The benchmarking for host DNA depletion was done using only 3 bacterial species, with just one Gram-positive. Including more broader pathogens would be beneficial for reaching conclusion.

4.----Blood culture positive samples usually have higher biomass appx 107 - 109 CFU/mL. It is expected that enough DNA would be extracted to use directly for nanopore sequencing. However, the authors have used amplification before sequencing. Can the authors discuss about the DNA yield obtained and the need for amplification?

5.----The number of clinical samples tested is limited to reach a conclusion for rapid diagnosis.

6.----AMR prediction is limited as only ResFinder is used.

7.----The overall mean read lengths are lower than expected for MDA samples. What is the rationale for using 0.5X beads for purification.

8.----Did the authors benchmark and see how the time for debranching with the T7 endonuclease would affect the read lengths? Since the 10 minutes used in the study seem to be high.

9.----Using the ligation kit for the clinical samples' validation seems to be interesting. However, the authors should clearly discuss the limitations regarding the cost associated with the ligation kit.

10. The statistics section is incomplete. They used R packages for visualization, but not for statistical methods.

Reviewer #1 (Comments for the Author):

In the manuscript "Rapid and modular workflows for same-day sequencing-based detection of bloodstream infections and antimicrobial resistance determinants using culture-enriched samples", Sajib et al. present the development of a workflow to detect bacterial species and AMR determinants in metagenomic sequencing data from blood samples, including thorough validation of various steps such as host depletion, incubation-based enrichment and sequencing library preparation. This is an impressive manuscript which provides detailed descriptions and justification of the methods used (including in the supplementary methods). Although long, I believe the level of detail provided in the manuscript is valuable for others working in the field of clinical metagenomics.

The aims and methods used in the study are valid, the introduction is well-written, and the discussion applies a suitable level of realistic criticism of the current applicability of the workflows for clinical diagnostics. Bearing in mind the previous revisions, I have no additional comments.

Answer: We thank Reviewer 1 for their positive and encouraging assessment of our manuscript.

Reviewer #2 (Comments for the Author):

1. How was fold depletion calculated? Although it is written in the figure legends, it should be mentioned in the methods section.

Answer: Thank you for this comment. We have now added a brief description in the method section. (Lines 121 – 123)

2. The statistical significance between the host depletion is missing.

Answer: As per the reviewer's suggestion, we have updated Table 1 and have added the paired t-test results. We have also briefly mentioned it in the methods section.
(Lines 123– 125 and Table 1)

3. The benchmarking for host DNA depletion was done using only 3 bacterial species, with just one Gram-positive. Including more broader pathogens would be beneficial for reaching conclusion.

Answer: We thank the reviewer for this constructive comment. These three bacterial species were used to initially benchmark the methods. We agree that broader pathogen inclusion would strengthen the conclusions and would aim to include additional species for further validation in future benchmarking.

4. Blood culture positive samples usually have higher biomass appx 107 - 109 CFU/mL. It is expected that enough DNA would be extracted to use directly for nanopore sequencing. However, the authors have used amplification before sequencing. Can the authors discuss about the DNA yield obtained and the need for amplification?

Answer: Thanks, although blood culture–positive broths are expected to contain sufficient bacterial biomass to support direct nanopore sequencing, this was not the approach used in our workflow. Following chemical host DNA depletion, we deliberately resuspended and diluted the depleted pellet in PBS. This step was included because host depletion with M-15, while highly efficient, cannot eliminate all host cells, and dilution further reduces the likelihood that residual host cells are carried forward into DNA extraction. From this diluted suspension, only 4 µL was taken forward for downstream processing. Consequently, the absolute DNA input available for library preparation was insufficient for direct sequencing.

5. The number of clinical samples tested is limited to reach a conclusion for rapid diagnosis.

Answer: We thank the reviewer for this comment. This issue was raised as a major point by previous reviewers at JCM, and we have therefore explicitly acknowledged this limitation in multiple sections of the revised manuscript submitted initially. Specifically, the limited number of clinical samples and the proof-of-concept nature of the study are discussed in the Abstract (Lines 48-49), Discussion (lines 533–534) and Limitations/Future directions (lines 571–573).

6. AMR prediction is limited as only ResFinder is used.

Answer: We agree that AMR prediction based on a single tool has inherent limitations. As discussed in the manuscript, this limitation is not specific to ResFinder but reflects a broader challenge in genotype-to-phenotype prediction, where the mere presence or absence of resistance genes or mutations does not always reliably predict phenotypic susceptibility.

We explicitly described this issue in the Discussion section (lines 586–592), where we note that alternative databases or search algorithms alone would not fully resolve this limitation. We further discuss that hybrid approaches, including the integration of multiple AMR databases, complementary algorithms, or machine-learning–based models, may improve predictive accuracy in the future. However, the development and benchmarking of such approaches, and the large, curated datasets required for them, are beyond the scope of this proof-of-concept study.

7. The overall mean read lengths are lower than expected for MDA samples. What is the rationale for using 0.5X beads for purification.

Answer: Interesting observation. Following MDA, our samples had an average fragment length of ~8–9 kb. Subsequent debranching reduced the effective fragment length distribution (data not shown). Also, for a subset of samples, additional shortening was introduced by use of the ONT Rapid Barcoding Kit, which employs a transposase-based barcoding mechanism (data not shown).

We applied a low-ratio SPRI bead clean-up (0.5×) to introduce size selection and reduce carry-over of short, fragmented DNA molecules into library preparation. This ratio closely aligns with the 0.6× bead clean-up recommended by NEB for debranching and clean-up of whole-genome amplification products (https://www.neb.com/en-gb/protocols/debranching-wga-rca-reaction-products-using-t7-endonuclease-i-m0302). Initially, we found that 0.6x did not consistently remove all short DNA fragments to the extent desired, and therefore we slightly reduced it to 0.5x to improve the final library size distribution (data not shown).

8. Did the authors benchmark and see how the time for debranching with the T7 endonuclease would affect the read lengths? Since the 10 minutes used in the study seem to be high.

Answer: We thank the reviewer for this comment. The manufacturer's recommended incubation time for T7 Endonuclease I–mediated debranching of WGA products is 60 minutes at 37 °C (https://www.neb.com/en-gb/protocols/debranching-wga-rca-reaction-products-using-t7-endonuclease-i-m0302). Although we did not systematically benchmark different incubation times against read length in this study, we explored shorter incubation durations in preliminary iterations (data not shown) and found that a 10-minute incubation reasonably debranched MDA products (note: can lead to 20-30% size reduction) while substantially reducing overall processing time, which was a key goal of our workflow.

9. Using the ligation kit for the clinical samples' validation seems to be interesting. However, the authors should clearly discuss the limitations regarding the cost associated with the ligation kit.

Answer: The cost implications of using a ligation-based library preparation workflow were already briefly mentioned in the Discussion; however, we have now slightly revised this section to make this limitation

more explicit. Specifically, we clarify that while the ligation sequencing kit produced the best output for clinical sample validation, it requires additional hands-on time and potentially pricier add-on reagents for library preparation (e.g. NEBNext Companion Module for Oxford Nanopore Technologies).

(Lines 508-509)

10. The statistics section is incomplete. They used R packages for visualization, but not for statistical methods.

Answer: Thanks for highlighting this. We have now revised this section by renaming it to “Statistical analysis and visualisation” and updating the relevant line numbers accordingly. In addition, we have expanded the text to explicitly describe that statistical tests were performed using base R functions, while additional R packages were used for data processing and visualisation.

(Lines 227-230)

Re: Spectrum03240-25R1 (Rapid and modular workflows for same-day sequencing-based detection of bloodstream infections and antimicrobial resistance determinants using culture-enriched samples)

Dear Dr. Mohammad Saiful Islam Sajib:

Your manuscript has been accepted, and I am forwarding it to the ASM production staff for publication. Your paper will first be checked to make sure all elements meet the technical requirements. ASM staff will contact you if anything needs to be revised before copyediting and production can begin. Otherwise, you will be notified when your proofs are ready to be viewed.

Data Availability: ASM policy requires that data be available to the public upon online posting of the article, so please verify all links to sequence records, if present, and make sure that each number retrieves the full record of the data. If a new accession number is not linked or a link is broken, provide production staff with the correct URL for the record. If the accession numbers for new data are not publicly accessible before the expected online posting of the article, publication may be delayed; please contact ASM production staff immediately with the expected release date.

Publication Fees: For information on publication fees and which article types have charges, please visit our website. We have partnered with Copyright Clearance Center (CCC) to collect author charges. If fees apply to your paper, you will receive a message from no-reply@copyright.com with further instructions. For questions related to paying charges through RightsLink, please contact CCC at ASM_Support@copyright.com or toll free at +1-877-622-5543. CCC makes every attempt to respond to all emails within 24 hours.

ASM Membership: Corresponding authors may join or renew ASM membership to obtain discounts on publication fees. Need to upgrade your membership level? Please contact Customer Service at Service@asmusa.org.

Embargo Policy: A press release may be issued as soon as the manuscript is posted on the Spectrum Latest Articles webpage. The corresponding author will receive an email with the subject line "ASM Journals Author Services Notification" when the article is available online.

The ASM Journals program strives for constant improvement in our submission and publication process. Please tell us how we can improve your experience by taking this quick Author Survey.

Thank you for submitting your paper to Spectrum.

Sincerely,
Sadjia Bekal
Editor
Microbiology Spectrum

Reviewer #2 (Comments for the Author):

All the comments have been addressed.
